# Instantaneous visual genotyping and facile site-specific transgenesis via CRISPR-Cas9 and phiC31 integrase

**DOI:** 10.1242/bio.061666

**Published:** 2024-09-03

**Authors:** Junyan Ma, Weiting Zhang, Simin Rahimialiabadi, Nikkitha Umesh Ganesh, Zhengwang Sun, Saba Parvez, Randall T. Peterson, Jing-Ruey Joanna Yeh

**Affiliations:** ^1^ Department of Basic Medical Science, Quanzhou Medical College, Quanzhou, Fujian 362011, China; ^2^ Cardiovascular Research Center, Massachusetts General Hospital, Charlestown, MA 02129, USA and Department of Medicine, Harvard Medical School, Boston, MA 02115, USA; ^3^ Department of Pharmacology and Toxicology, University of Utah, Salt Lake City, UT 84112, USA; ^4^ Center for Immunology and Inflammatory Disease, Massachusetts General Hospital, Charlestown, MA 02129, USA

**Keywords:** CRISPR, Cas9, PhiC31, Integrase, Targeted integration, Genotyping, Transgenesis, Reporter, Zebrafish, Knock-in

## Abstract

Here, we introduce ‘TICIT’, targeted integration by CRISPR-Cas9 and integrase technologies, which utilizes the site-specific DNA recombinase – phiC31 integrase – to insert large DNA fragments into CRISPR-Cas9 target loci. This technique, which relies on first knocking in a 39-basepair phiC31 landing site via CRISPR-Cas9, enables researchers to repeatedly perform site-specific transgenesis at the exact genomic location with high precision and efficiency. We applied this approach to devise a method for the instantaneous determination of a zebrafish's genotype simply by examining its color. When a zebrafish mutant line must be propagated as heterozygotes due to homozygous lethality, employing this method allows facile identification of a population of homozygous mutant embryos even before the mutant phenotypes manifest. Thus, it should facilitate various downstream applications, such as large-scale chemical screens. We demonstrated that TICIT could also create reporter fish driven by an endogenous promoter. Further, we identified a landing site in the *tyrosinase* gene that could support transgene expression in a broad spectrum of tissue and cell types. In sum, TICIT enables site-specific DNA integration without requiring complex donor DNA construction. It can yield consistent transgene expression, facilitate diverse applications in zebrafish, and may be applicable to cells in culture and other model organisms.

## Teaser

**Summary:** Single-copy transgene integration is achieved at user-specified genomic loci to facilitate multiple downstream applications.

## INTRODUCTION

The zebrafish is a genetically tractable vertebrate animal model that has recently gained popularity in biomedical research (Choi et al., 2021; Demarest and Brooks-Kayal, 2018; Fazio et al., 2020; Gut et al., 2017; Rissone and Burgess, 2018; Sakai et al., 2018; Torraca and Mostowy, 2018). As the scientific community seeks to assign functions to thousands of human genetic variants being identified, the scale and throughput enabled by the zebrafish are particularly useful. Moreover, zebrafish models permit chemical screens guided by successful reversal of disease-related phenotypes in a whole organism, which may substantially reduce discovery time and attribution rate during the development of therapeutics (Helenius and Yeh, 2012; MacRae and Peterson, 2015; Swinney and Anthony, 2011). Hence, developing genome engineering tools to create zebrafish models more effectively and efficiently is expected to propel biomedical research that can later be translated into medicine (Cully, 2019; Patton et al., 2021).

While the zebrafish is highly amenable to genetic manipulations including Tol2-mediated transgenesis and CRISPR-Cas9-mediated mutations, the current methods have vexing shortcomings. Although transgenesis using the Tol2 transposon system is quite efficient, its outcome is often unpredictable and inconsistent between individual animals due to the variability in the number and location of integration event(s) (Abe et al., 2011). Thus, when using this method to compare the functions of different genetic variants, researchers often need to analyze multiple transgenic lines and outcross the animals for several generations in order to obtain reliable, consistent results. In contrast, in mice and other mammalian models, Rosa26 has been extremely useful as a ‘safe harbor’ genomic locus enabling ubiquitous transgene expression or faithful control of transgene expression by its own promoter (Chen et al., 2011; Kong et al., 2014; Soriano, 1999). Single copy integration at the Rosa26 locus can be achieved by homologous recombination in embryonic stem cells or mouse zygotes (Meyer et al., 2010; Soriano, 1999). Nonetheless, to date, a defined safe harbor genomic locus has not been widely recognized in zebrafish. Notably, various methods for targeted integration of large DNA fragments in zebrafish have been developed via homologous recombination, microhomology-mediated end joining (MMEJ), or homology-independent mechanisms (Auer et al., 2013; DiNapoli et al., 2020; Hisano et al., 2015; Hoshijima et al., 2019; Shin et al., 2014; Wierson et al., 2020; Zhang et al., 2016). However, in addition to their low to moderate knock-in efficiencies, these techniques may also lead to high rates of mutations and imprecise integrations at the target sites (Auer et al., 2013; DiNapoli et al., 2020; Hisano et al., 2015; Hoshijima et al., 2019; Shin et al., 2014; Wierson et al., 2020; Zhang et al., 2016). Innovations and tools that allow zebrafish researchers to choose a genomic location of interest and exploit it for facile transgenesis are still lacking.

The DNA integrase of phiC31 bacteriophage is a site-specific recombinase that mediates DNA recombination between two heterotypic binding sequences named attB and attP, which are converted into two attB/attP hybrid sequences, termed attL and attR, after the recombination (Hillman and Calos, 2012). This reaction is irreversible, and thus phiC31 recombinase can mediate stable integration (Hillman and Calos, 2012). Moreover, phiC31-mediated integration does not require any cellular auxiliary factors (Thorpe and Smith, 1998). It has been successfully used to insert large DNA constructs (10-100 kb) into the genomes of mammalian cells, fruit flies, *Xenopus*, and zebrafish (Belteki et al., 2003; Bischof et al., 2007; Li et al., 2012; Mosimann et al., 2013; Roberts et al., 2014). Previously, Mosimann, et al. and Roberts, et al. generated phiC31 transgenesis recipient zebrafish lines via Tol2 and demonstrated that DNA vectors containing the attB sequence could be inserted into genomic attP sites, resulting in mean germline transmission efficiencies of 34% and 10% in the two studies (Mosimann et al., 2013; Roberts et al., 2014). Encouraged by their results, we explored additional applications using phiC31 integrase.

**Fig. 1.**
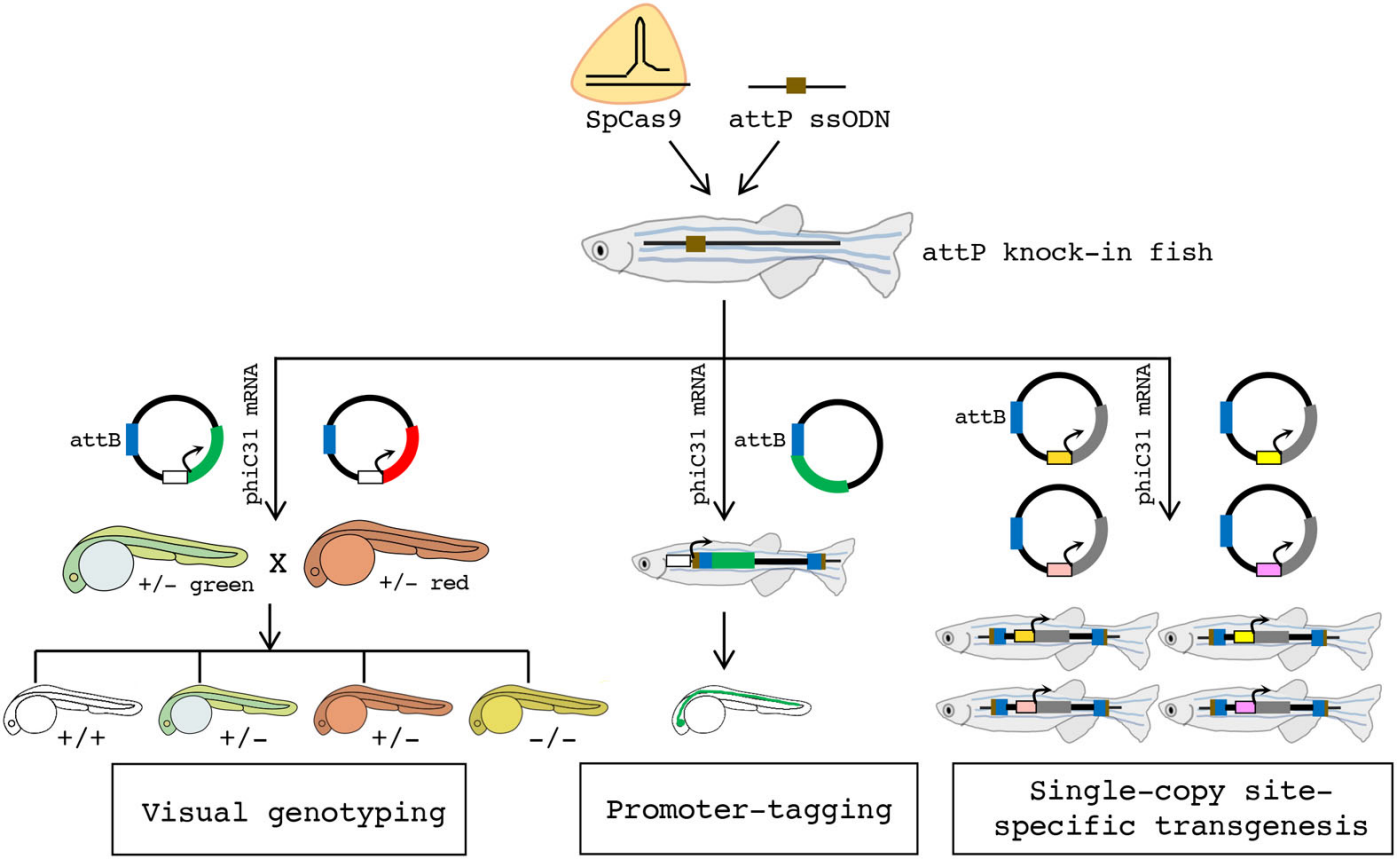
**An overview of the TICIT technology for visual genotyping, promoter-tagging, and single-copy, site-specific transgenesis.** CRISPR-Cas9 from *S. pyogenes* (SpCas9) is employed together with a single-stranded oligodeoxynucleotide (ssODN) containing the phiC31 landing site to create attP knock-in fish lines. Next, single-copy site-specific transgene integration is achieved by co-delivering the phiC31 mRNA with any plasmids containing the phiC31 attachment sequence attB. For visual genotyping, two plasmids carrying fluorescent markers driven by a promoter of user's choice (shown as unshaded boxes) are used. This enables Cas9-mediated gene mutations to be identified by fluorescence even before mutant phenotypes manifest. In a heterozygous intercross, wild-type offspring are unmarked, heterozygous offspring are either green or red, and homozygous offspring are both green and red (shown as yellow). For promoter-tagging, a plasmid carrying a promoter-less fluorescent marker adjacent to the attB sequence is used. After phiC31-mediated integration, the promoter activity of the target gene can be tracked by the expression of the fluorescent marker. Further, fish lines carrying attP knock-in at pre-defined loci may enable facile transgenesis devoid of positional and copy number artifacts.

This study combined CRISPR-Cas9 and phiC31 technologies and developed a workflow enabling facile single-copy, site-specific transgenesis at any user-specified genomic loci (Fig. 1). Using this method called targeted integration by CRISPR-Cas9 and integrase technologies or TICIT, researchers can pre-select a suitable, well-characterized genomic location for inserting their transgenes. Given that multiple transgenic lines can be generated with insertions occurring at the same locus, expression differences due to different insertion sites can be avoided, and the expression levels of the transgenes among different transgenic lines are expected to be similar. Meanwhile, compared to other homology-directed approaches, this method eliminates the need for constructing homology arms into transgene vectors for each target locus. Instead, any vectors that contain a 70-basepair attB sequence can be used for integration. Researchers can also use TICIT to manipulate their genes of interest. Here, we applied this method to generate allele-tracking markers that allow quick sorting of mutant embryos for any downstream applications (Fig. 1). We characterized a genomic locus that may be useful for future transgenesis studies. Finally, we showed that TICIT can mediate in-frame integration enabling transgene expression controlled by an endogenous promoter (Fig. 1).

## RESULTS

### Generation of genomic attP landing sites using CRISPR-Cas9

**Fig. 2.**
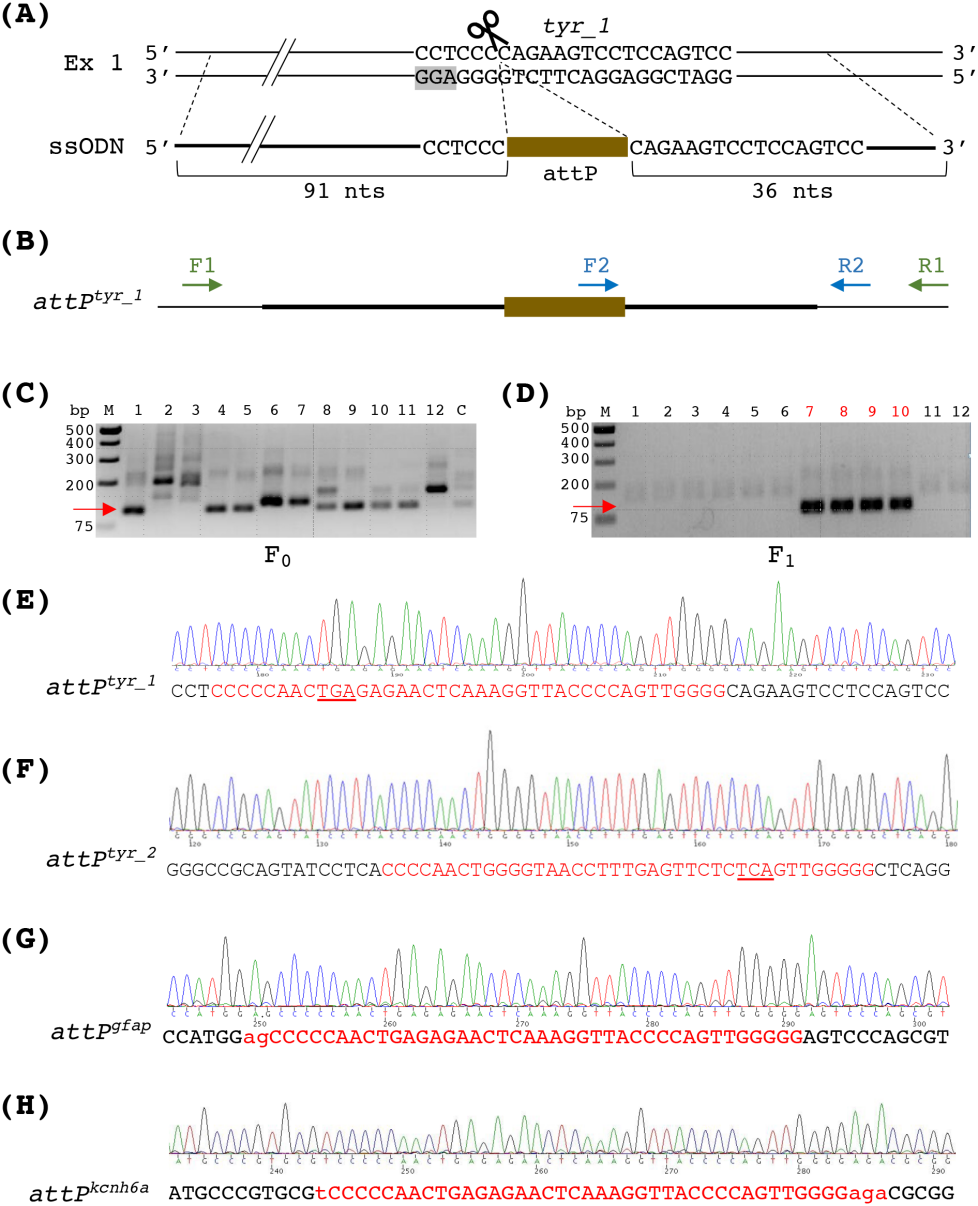
**Generation of the attP knock-in fish lines.** (A) Schematic of the SpCas9 target locus for *tyr_1* and its corresponding single-stranded oligonucleotide (ssODN). The *tyr_1* site is in the first exon (Ex 1) of the *tyrosinase* gene. Its target sequence is shown, and the spacer adjacent motif (PAM) is highlighted in gray. The cleavage site of SpCas9 is indicated by a pair of scissors. The ssODN used for attP knock-in harbors attP encompassed by two homology arms. The sequences for the homology arms were complementary to the non-target strand of SpCas9 and were 36 nts long on the PAM-distal side and 91 nts on the PAM-proximal side. Complete sequences of the ssODNs for attP knock-in at both *tyr_1* and *tyr_2* sites can be found in Table S4. (B) Schematic of the attP knock-in at the *tyr_1* locus (designated as the *attP^tyr_1^* allele) and the primers for PCR analysis. The thick black line indicates the overlapping sequence with the ssODN. To detect the attP insertion, nested PCR was performed using *tyr*-specific F1 and R1 primers, followed by F2 and R2 primers that recognize attP and the *tyr* gene, respectively. (C) Representative agarose gel analysis of the nested PCR from single embryos microinjected with SpCas9 protein, the *tyr_1* gRNA, and the ssODN. The expected product size is 110 bps as indicated by a red arrow. M, size marker; 1-12, injected embryos; C, an uninjected control embryo. (D) Representative agarose gel analysis of the nested PCR from the *attP^tyr_1^* founder screen. Twelve pools of embryos (five embryos per pool) from each potential founder were analyzed. The 110-bp band (red arrow) indicated the presence of the *attP^tyr_1^* allele. The pools containing the attP knock-in alleles are labeled by red numbers on top. (E-H). Sequences of the attP knock-in at the *tyr_1* (E), *tyr_2* (F), *gfap* (G), *and kcnh6a* (H) targeted loci in the F_1_ fish. Endogenous gene sequences are in black letters. Insertions are in red letters. The attP sequences are in uppercase. For *attP^tyr_1^* and *attP^tyr_2^*, in-frame stop codons are underlined. For *attP^gfap^* and *attP^kcnh6a^*, the lowercase letters are sequences added to keep the insertions in-frame for unperturbed translation of the endogenous proteins.

To insert attP into genomic target loci, we used *Streptococcus pyogenes* Cas9 (SpCas9) to create double-strand DNA breaks and used single-stranded oligodeoxynucleotides (ssODNs) as the donor DNA for DNA repair. This technique has been used to create designer mutations in zebrafish (Boel et al., 2018; Petri et al., 2022; Prykhozhij et al., 2018). Meanwhile, using this method, we have previously shown that small precise edits can be introduced at an allele frequency up to ∼10% in the injected embryos (Petri et al., 2022). We first targeted two SpCas9 cleavage sites in the *tyrosinase* (*tyr*) gene, named *tyr_1* and *tyr_2*. Both guide RNAs (gRNAs) can efficiently mutate *tyr* and elicit the albino phenotype (Jao et al., 2013; Moreno-Mateos et al., 2017). We designed the ssODNs to contain an attP flanked by two short homology arms adjacent to the SpCas9 cleavage sites (Fig. 2A and Table S4). For attP, a 39-basepair (bp) minimal sequence that showed full recombination activity in human cells and had little or no effect on transgene expression was used (Calos, 2006; Kirchmaier et al., 2013; Mosimann et al., 2013). The attP sequence was inserted in different orientations in the ssODNs for these two loci so that both knock-in alleles possessed an in-frame stop codon in *tyr*. Further, the sequences for the homology arms were complementary to the non-target strand of SpCas9. They were 36 nucleotides (nts) long on the protospacer adjacent motif (PAM)-distal side and 91 nts on the PAM-proximal side. We and others have previously shown that this donor DNA configuration is effective in zebrafish and human cells (Petri et al., 2022; Prykhozhij et al., 2018; Richardson et al., 2016). The ssODNs were chemically synthesized and two phosphorothioate linkages were added to both termini to enhance stability and knock-in efficiency (Prykhozhij et al., 2018).

We performed microinjection of 1-cell stage zebrafish embryos with SpCas9 and gRNA ribonucleoprotein (RNP) complexes and the ssODN for each sgRNA. Subsequently, successful knock-in events were identified in two ways. First, we conducted two-step nested PCR to detect the attP integration in the *tyr* gene. As illustrated in Fig. 2B for the *tyr_1* locus, a pair of *tyr*-specific primers (denoted as F1 and R1) located outside the region of the donor DNA was used for the first round of nested PCR to amplify the targeted locus. We used another *tyr*-specific primer (R2) and an attP-specific primer (F2) for the second-round PCR to amplify the attP knock-in alleles. This yielded PCR products of both expected and incorrect lengths, suggesting that some knock-in alleles may contain additional insertions or deletions (Fig. 2C). Second, PCR-positive samples were chosen, and their amplification products generated using a set of *tyr*-specific primers were subjected to next-generation sequencing. The results showed that this method had successfully inserted the entire attP site into both target loci (Table 1).

**Table 1.**
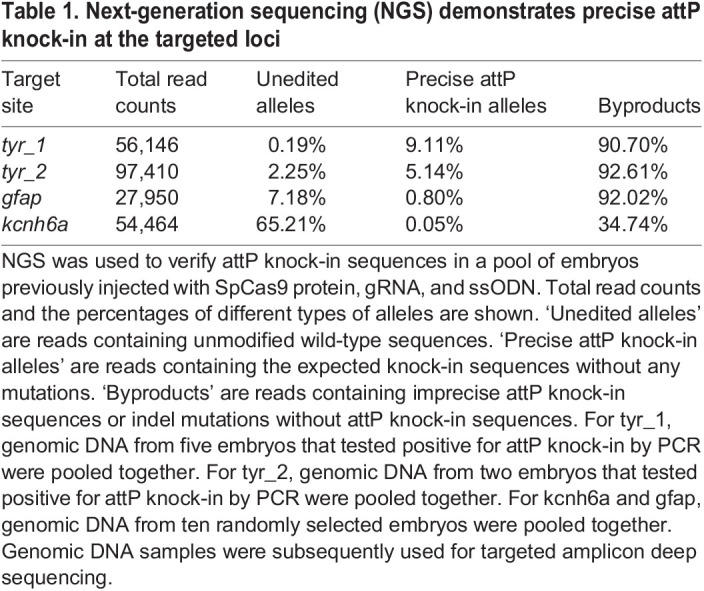
Next-generation sequencing (NGS) demonstrates precise attP knock-in at the targeted loci

We raised the injected embryos to adulthood, outcrossed them to wild-type fish, and screened for founders. The same nested PCR strategy was employed to identify the embryos carrying the attP knock-in alleles (Fig. 2D). Further, the attP knock-in sequences in the F_1_ embryos were verified via Sanger sequencing (Fig. 2E,F). For *tyr_1*, we identified one founder from 16 F_0_ fish screened. For *tyr_2*, we identified one founder from three F_0_ fish screened. Thus, founder frequencies were 6.3% and 33.3% for *tyr_1* and *tyr_2*, respectively. Germline mosaicism of the identified founders was 5.5-11.9% (Table 2). When F_1_ fish reached adulthood, we identified heterozygous fish carrying the attP knock-in alleles (hereafter named the *attP^tyr_1^* and *attP^tyr_2^* alleles) by fin clipping and PCR. We incrossed heterozygous fish for both lines and found that approximately 25% of their offspring showed the albino phenotype (data not shown), indicating that both attP insertions disrupted the *tyr* gene as expected. Together, these results indicate that we have established *tyr* mutant lines with the phiC31 landing site in the pre-defined *tyr* loci.

**Table 2.**
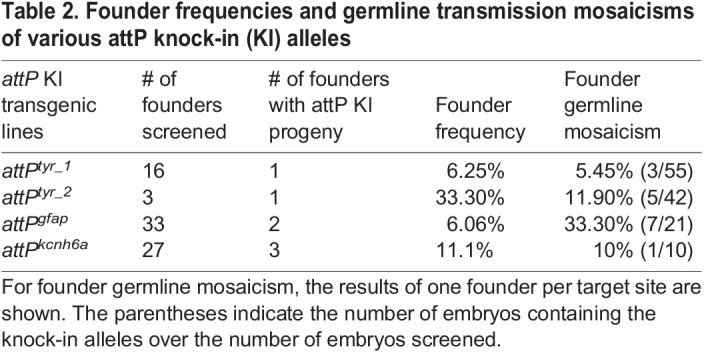
Founder frequencies and germline transmission mosaicisms of various attP knock-in (KI) alleles

To evaluate the robustness of the attP knock-in method, we sought to insert attP into two more genes – the *glial fibrillary acidic protein* (*gfap*) gene and the *potassium voltage-gated channel, subfamily H, member 6a* (*kcnh6a*) gene. We designed and tested two to three gRNAs for each gene and identified one with 57% mutation efficiency for *gfap* and another one with 38.6% mutation efficiency for *kcnh6a* based on the PCR-fluorescent fragment length analysis (Table S5). Next, we designed ssODNs to contain an attP encompassed by two short homology arms as described above, except that we avoided having a premature stop codon in the knock-in alleles of these genes (Table S4). We performed microinjection of SpCas9 protein, gRNA, and ssODN, and we detected successful attP knock-in in the microinjected embryos via next-generation sequencing (Table 1). Following similar genotyping strategies used for the *tyr* loci, we identified two *attP^gfap^* founders from 33 F_0_ fish screened and three *attP^kcnh6a^* founders from 27 F_0_ fish screened (Table 2). Thus, founder frequencies for *attP^gfap^* and *attP^kcnh6a^* were 6.1% and 11.1%, respectively. The knock-in sequences were verified (Fig. 2G,H), and the germline mosaicism was 10-33% for the founders (Table 2). In sum, we have successfully created phiC31 transgenesis recipient lines in multiple zebrafish genes.

### Generation of allele-tracking reporter lines using phiC31 integrase

**Fig. 3.**
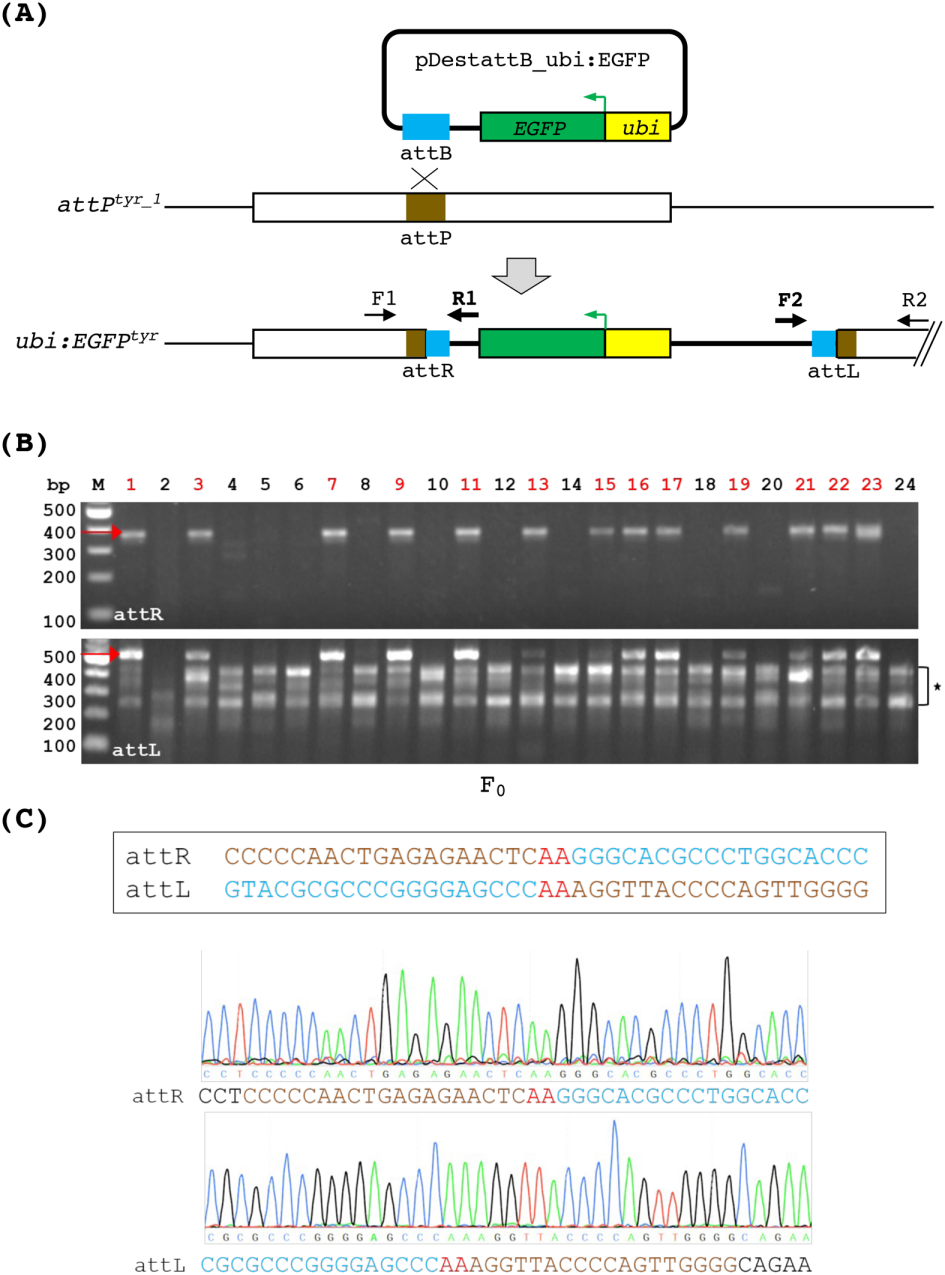
**PhiC31 integrase mediates DNA integration via recombination between attB and the genomic attP site.** (A) A diagram depicting the integration of *ubi:EGFP* at the *attP^tyr_1^* locus. PhiC31 integrase mediates recombination between the plasmid DNA containing the attB sequence and the genomic attP site, resulting in single-copy integration of the entire plasmid. The integrated DNA (thick black line) is flanked by two attP/attB composite sequences named attR and attL, which can be detected via PCR using two sets of primers (F1/R1 and F2/R2) as indicated. F1 and R2 are *tyr*-specific, whereas R1 and F2 are plasmid-specific. (B) PCR analysis of attR and attL in individual embryos microinjected with the phiC31 mRNA and pDestattB_ubi:EGFP. Red arrows indicate the expected product sizes of attR (369 bps, top panel) and attL (534 bps, bottom panel). The bracket with an asterisk on the right side of the bottom gel indicates non-specific amplifications from the F2/R2 primer set. The embryos with the correct size products from both attR and attL PCR are indicated by red numbers on the top. (C) Sanger sequencing of the attR (top chromatogram) and attL (bottom chromatogram) PCR products demonstrates precise DNA integration. Reference sequences for attR and attL are shown in the box above the chromatograms. Sequences originating from attP and attB are indicated in brown and turquoise, respectively. The ‘AA’ crossover site is shown in red.

Theoretically, an attP site located in an endogenous gene will enable the facile generation of a fluorescently tagged mutant allele via the phiC31 integrase technology, eliminating the need for time-consuming genotyping procedures. To test this, we mated heterozygous *attP^tyr_1^* fish to wild-type fish. We microinjected their embryos with *in vitro* transcribed phiC31 mRNA and the plasmid pDestattB_ubi:EGFP originally developed by Mosimann et al. (Mosimann et al., 2013). This plasmid contains a 70-bp attB sequence and the *EGFP* gene driven by the *ubiquitin* (*ubi*) promoter, which can elicit ubiquitous green fluorescence from an early embryonic stage (Mosimann et al., 2011). We used two sets of PCR primers to detect the 5′ and 3′ ends of phiC31-mediated integration in the injected embryos (Fig. 3A). The data showed that 13 out of 24 analyzed embryos exhibited correct integration at both 5′ and 3′ ends (Fig. 3B). Since it was expected that only half of the embryos would carry the attP knock-in allele, these results suggest that phiC31-mediated DNA recombination was very efficient at the *attP^tyr_1^* locus. We performed the same test using heterozygous *attP^tyr_2^* fish. However, PCR analysis did not detect any integration events, suggesting that the *attP^tyr_2^* locus may be defective or inaccessible for phiC31 integrase (data not shown). Consequently, we chose *attP^tyr_1^* for the following study. Sanger sequencing results confirmed the attL and attR sequences flanking the integration of pDestattB_ubi:EGFP at the *attP^tyr_1^* locus, indicating precise recombination between attB and attP (Fig. 3C). Taken together, these results demonstrate that phiC31 integrase can mediate precise and efficient DNA integration in zebrafish.

**Fig. 4.**
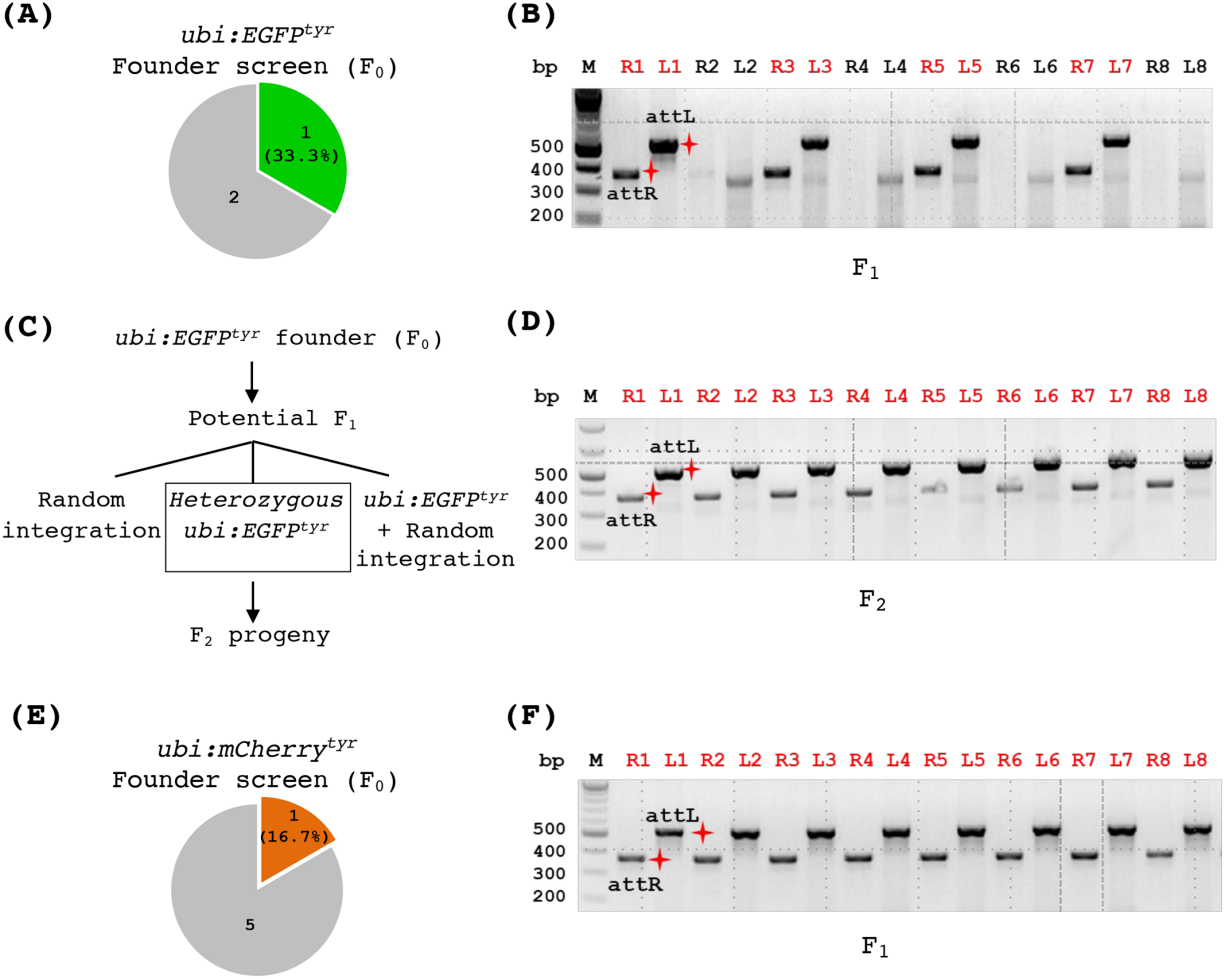
**Generation of allele-tracking reporter lines for the *tyr* mutation.** (A,B) Founder screen results of the transgenic *ubi:EGFP^tyr^* zebrafish. (A) A pie chart indicating the number of F_0_ fish screened that produced some fluorescent embryos (the green piece) or all non-fluorescent embryos (the gray piece). (B) PCR analysis of individual fluorescent F_1_ embryos demonstrating that some of them (labeled by red numbers on top) carried a correct *ubi:EGFP* integration. ‘R’ and ‘L’ in front of the numbers on top indicate attR and attL PCR, respectively. The red shining star symbols mark the correct size products of attR (369 bps) and attL (534 bps). (C) Schematic depicting potential genotypes of the descendants from an *ubi:EGFP^tyr^* founder fish. (D) PCR analysis of individual fluorescent embryos (F_2_) from a heterozygous *ubi:EGFP^tyr^* fish (F_1_) indicating that all of them carried the correct integration. (E,F) Founder screen results of the transgenic *ubi:mCherry^tyr^*zebrafish. (E) A pie chart indicating the number of F_0_ fish screened that produced some fluorescent embryos (the red piece) or all non-fluorescent embryos (the gray piece). (F) PCR analysis of individual fluorescent F_1_ embryos showing that all of them carried a correct *ubi:mCherry* integration.

To evaluate germline transmission of the recombinant allele, we raised the injected embryos from heterozygous *attP^tyr_1^* outcrosses to adulthood. We screened three fish and identified one founder that produced green-fluorescent offspring (Fig. 4A). The ratio between green and non-green F_1_ embryos was approximately 1:1. Using PCR, we could detect the correct integration of *ubi:EGFP* at the *attP^tyr_1^* locus in 50% of the green fluorescent embryos, suggesting that this founder carried not only phiC31-mediated but also random integration (Fig. 4B). This result is unsurprising since random integration can occur at low frequency from plasmid DNA microinjection. It should be noted that random integration has been observed less frequently compared to phiC31-mediated integration into transgenic attP sites in zebrafish, and there are presently no known functional pseudo-attP sites in the zebrafish genome (Mosimann et al., 2013). We raised green-fluorescent F_1_ fish to adulthood and performed fin-clipping and PCR to identify the F_1_ fish that harbored the correct *ubi:EGFP* integration. Since these fish could still carry more than one *EGFP* integration (Fig. 4C), we outcrossed them and selected the ones that produced approximately 1:1 of green versus non-green embryos. Further, we verified that all fluorescent F2 progeny we analyzed carried the correct integration by PCR (Fig. 4D). These results indicate that we have successfully generated heterozygous fish carrying a single-copy, *ubi:EGFP* reporter in the *tyr* gene (denoted as the *ubi:EGFP^tyr^* allele).

A similar workflow was carried out for generating a red fluorescent report line to track the *tyr* mutant allele. We generated the pDestattB_ubi:mCherry construct and injected it with the phiC31 mRNA into the embryos of heterozygous *attP^tyr_1^* fish outcrosses. When the injected embryos reached adulthood, we screened six F_0_ fish that exhibited high mCherry mosaicism and identified one founder (Fig. 4E). This founder fish produced approximately 50% red fluorescent progeny when crossed with a wild-type fish. Moreover, all fluorescent embryos analyzed by PCR showed a correct integration (Fig. 4F). We raised red fluorescent F_1_ fish to adulthood, outcrossed three fish to the wild-type fish, and found that all three carried a single-copy integration of *ubi:mCherry* at the *tyr* gene. Overall, these results demonstrate that attP fish lines are useful transgenesis recipients. One attP fish line can be used to derive multiple integration lines with ease. Meanwhile, phiC31 integrase can mediate efficient and transmissible DNA integration at genomic attP landing sites.

### Instantaneous visual genotyping using the *tyr* allele-tracking reporter lines

**Fig. 5.**
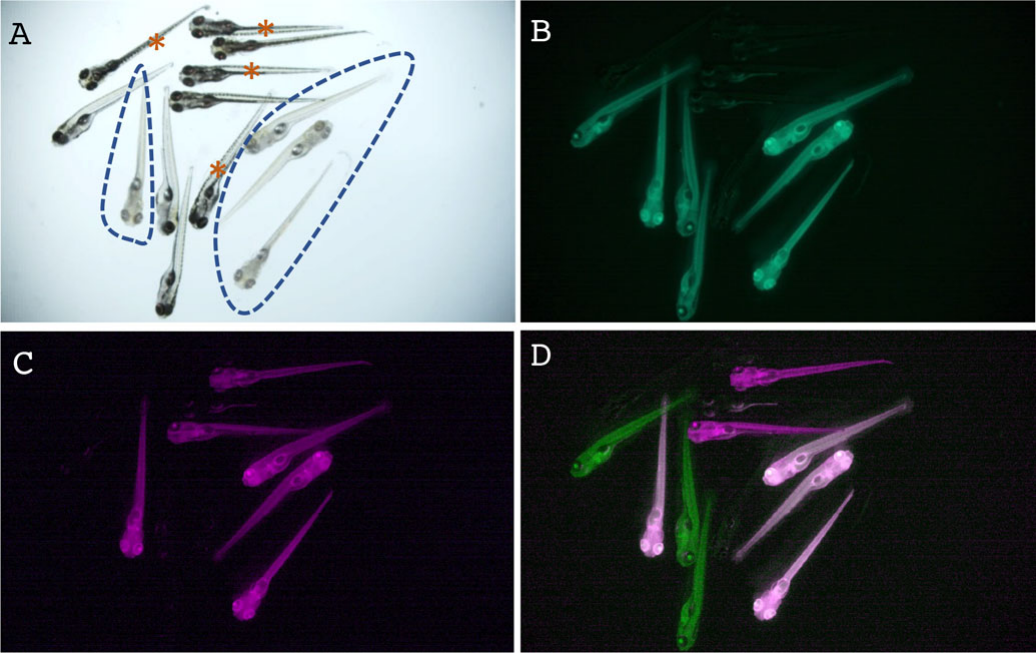
**Instantaneous visual genotyping using the *tyr* allele-tracking reporter lines.** (A-D) Visual genotyping results of the *tyr* mutation using *ubi:EGFP^tyr^* and *ubi:mCherry^tyr^*, two allele-tracking reporter lines. Heterozygous fish of these two lines were intercrossed, and their offspring were inspected under a fluorescent microscope. The same group of embryos was imaged in the bright field (A), green-fluorescent channel (B, shown in green), and red-fluorescent channel (C, shown in magenta). Images of B and C were merged in D. In panel A, embryos exhibiting the homozygous *tyr* mutant phenotype, as evidenced by the loss of pigmentation, were encircled by dashed lines. In panel D, embryos that were both green and magenta were white. Embryos that had no fluorescence in B to D were indicated by brown asterisks in A. Hence, the wild-type embryos were unmarked, the heterozygous embryos were either green or magenta, and the homozygous embryos were both green and magenta.

Having created the allele-tracking reporter lines with two different colors for the *tyr* gene, we sought to demonstrate the feasibility of a technique for instantaneous visual genotyping. In this method, by mating two heterozygous mutant fish that each has its own marker linked to the same mutation, one can determine whether a progeny is a wild type, heterozygote, or homozygote simply by examining its color. Hence, we mated a heterozygous *ubi:EGFP^tyr^* fish to a heterozygous *ubi:mCherry^tyr^* fish and found that all double fluorescent fish exhibited the albino phenotype and vice versa (Fig. 5). Thus, these results demonstrate the advantages of constructing mutant lines along with allele-tracking reporters via the combination of CRISPR-Cas9 and phiC31 technologies.

### Broad transgene expression at the *attP^tyr^* landing site

**Fig. 6.**
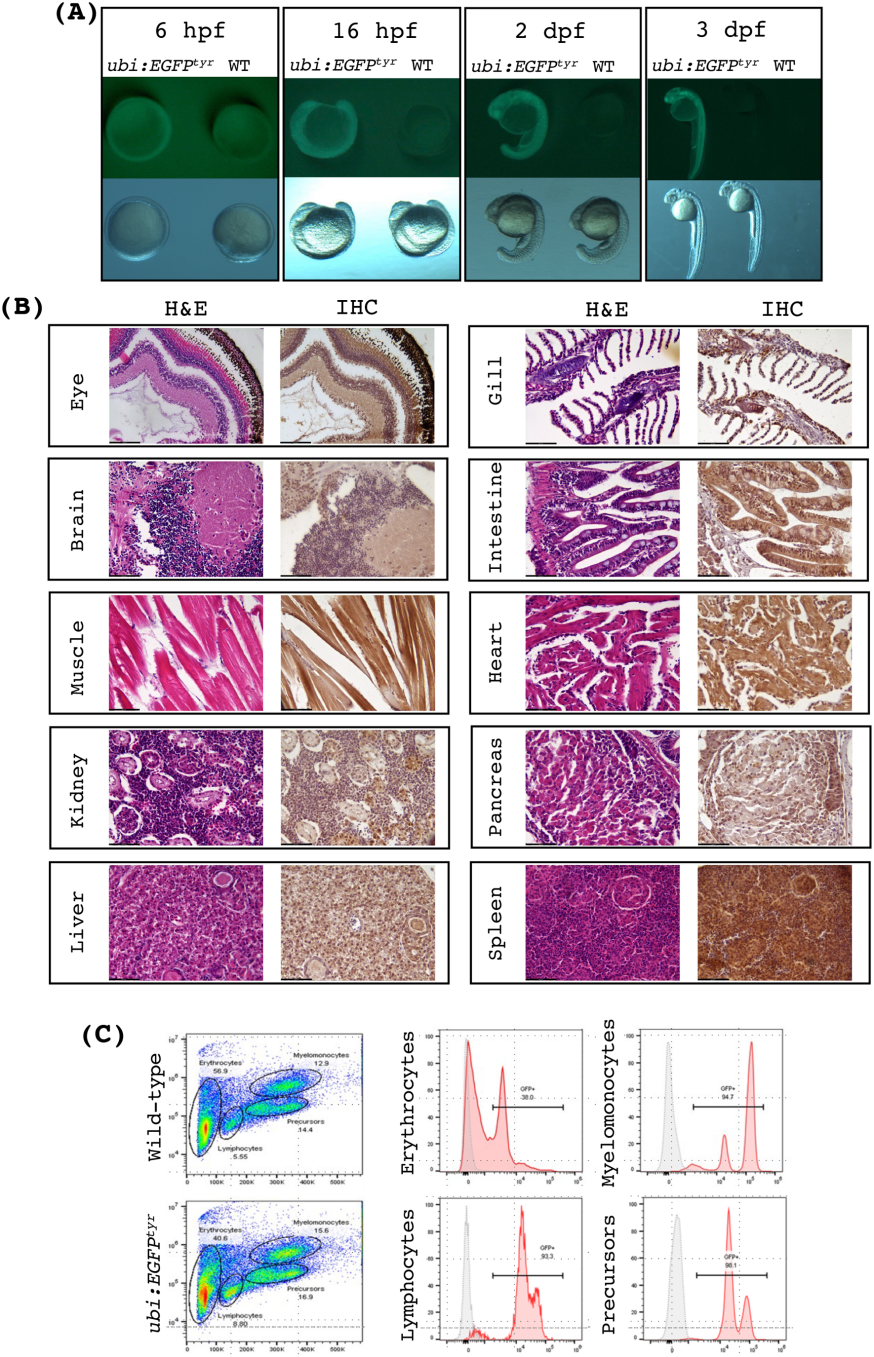
***ubi:EGFP^tyr^* transgenic fish exhibit robust and ubiquitous EGFP expression.** (A) Bright-field and fluorescence images of *ubi:EGFP^tyr^* embryos versus wide-type (WT) at various developmental stages as indicated. Each panel contains an *ubi:EGFP^tyr^* embryo on the left and a WT embryo on the right. hpf, hours post fertilization; dpf, days post fertilization. (B) Histology sections of *ubi:EGFP^tyr^* adult fish stained with H&E or anti-EGFP antibody (IHC) demonstrating widespread but varying levels of EGFP expression among different tissue and cell types. All images were acquired under 400X magnification. (C) Representative flow cytometry analysis of the wild-type and *ubi:EGFP^tyr^* adult whole kidney marrow (WKM). Major blood lineages in WKM were resolved and identified as previously shown (left panels).^36^ EGFP expression was analyzed in each blood cell gate (middle and right panels). Gray, wild-type; pink, *ubi:EGFP^tyr^*.

In the *ubi:EGFP^tyr^* fish, we observed broad and strong green fluorescence from an early embryonic stage, suggesting that the *attP^tyr^* landing site could support transgene expression in multiple tissues. To investigate this further, we examined the fluorescence of heterozygous *ubi:EGFP^tyr^* fish from embryonic to adult stages. Using a fluorescent stereoscope, we could see faint EGFP expression starting 6 h post fertilization (hpf) (Fig. 6A). The intensity of green fluorescence soon became apparent, appeared ubiquitous, and continued throughout the embryonic stages (Fig. 6A). All heterozygous *ubi:EGFP^tyr^* adult fish showed consistently strong and ubiquitous green fluorescence from outside (Fig. S1A). We dissected the adult fish and found that *EGFP* was expressed in all organs and tissue types, such as muscle, gill, eye, brain, skin, spleen, heart, liver, kidney, intestine, pancreas, testis, and ovary (Fig. S1B). However, the fluorescence was noticeably absent in mature eggs, which was different from another *ubi* reporter line (*ubi:loxP-EGFP-loxP-mCherry*, also known as *ubi:Switch*) that showed maternally deposited *EGFP* expression (Fig. S1C) (Mosimann et al., 2011). Next, to examine some of the tissue and cell types more closely, we performed immunohistochemistry (IHC) using an anti-GFP antibody. The results showed that EGFP could be detected in all tissue sections, even though it was not at the same level among different cell types (Fig. 6B and Fig. S2). These data are expected as it was previously shown that the *ubi* promoter renders a ‘ubiquitous’, but not necessarily ‘homogenous’ expression in all cell types (Mosimann et al., 2011). On the other hand, we cannot preclude the possibility that the heterogenous levels of *GFP* expression might also be due to a potential interaction between the *tyr_1* locus and the integrated *ubi* promoter.

Further, we isolated hematopoietic cells from the whole kidney marrow, the hemogenic tissue in zebrafish, and analyzed the fluorescence of various blood lineages using flow cytometry (Fig. 6C). We found that *EGFP* was expressed in >90% of hematopoietic progenitors, lymphocytes, and myelomonocytes and ∼40% of erythrocytes. Taken together, these findings demonstrate that the *attP^tyr^* landing site can support transgene expression in a wide range of tissue and cell types.

### Generation of a promoter-tagging line via TICIT

**Fig. 7.**
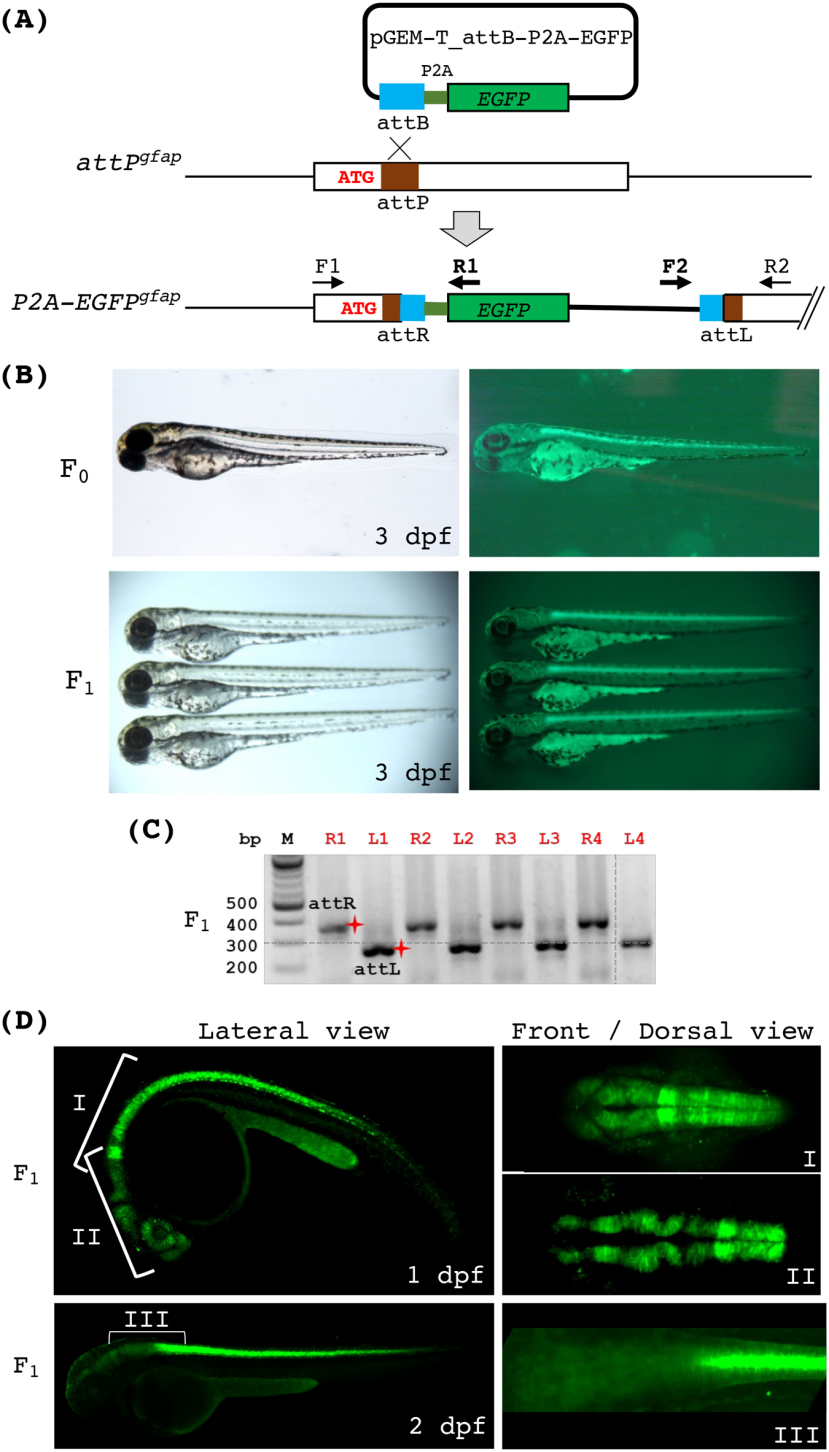
**Generation of the endogenous *gfap* reporter line via phiC31 integrase.** (A) A diagram depicting the integration of a promoter-less EGFP reporter at the *attP^gfap^* locus via phiC31-mediated recombination. Correct integration of pGEM-T_attB-P2A-EGFP will result in fusion and co-translation of the N-terminus of gfap and P2A-EGFP. We named this allele *P2A-EGFP^gfap^*. Two sets of PCR primers (F1/R1 and F2/R2 as indicated) were used to detect the integration. F1 and R2 are *gfap*-specific, whereas R1 and F2 are plasmid-specific. (B) Bright-field and fluorescence images of endogenous *gfap* reporter fish embryos at 3 dpf. The top panels show an *attP^gfap^* embryo after microinjection with the phiC31 mRNA and pGEM-T_attB-P2A-EGFP. The bottom panels show three heterozygous *P2A-EGFP^gfap^* embryos. (C) PCR analysis of individual fluorescent embryos from a founder fish indicating that all of them harbored the correct integration. ‘R’ and ‘L’ in front of the numbers on top indicate attR and attLPCR, respectively. The red shining star symbols mark the correct size products of attR (332 bps) and attL (282 bps). (D) Stacked confocal images of the *P2A-EGFP^gfap^* embryos showing various regions and angles of view at 1 and 2 dpf.

Another potential application of single-copy transgenesis at a user-specified genomic location is to generate gene-tagging or promoter-tagging lines. We sought to insert a reporter gene into the *attP^gfap^* locus as a proof of concept. During embryonic development, *gfap* is abundantly expressed in the glial cells of the eye and the central nervous system (CNS). To do this, we first generated a plasmid pGEM-T_P2A-EGFP by inserting the coding sequences of the self-cleaving peptide P2A and a promoter-less *EGFP* gene next to the attB site in pGEM-T (see the Materials and Methods). We expected that phiC31-mediated recombination between the plasmid DNA and the genomic *attP^gfap^* locus should result in an in-frame integration of *EGFP* after the start codon of *gfap* (Fig. 7A). Thus, we microinjected pGEM-T_P2A-EGFP and the phiC31 mRNA into the embryos of heterozygous *attP^gfap^* fish outcrossed to the wild-type fish. In the injected embryos, we could readily see mosaic *EGFP* expression, specifically in the CNS (Fig. 7B). When the injected embryos reached adulthood, we screened one F_0_ fish with *EGFP* expression in the CNS and found that it produced 18% fluorescent progeny (32 out of 175 embryos). All F_1_ fluorescent embryos expressed a consistent level and pattern of *EGFP* expression in the CNS (Fig. 7B), and all fluorescent embryos analyzed by PCR showed a correct integration (Fig. 7C). Further, confocal imaging analysis showed EGFP expression in the eye and the brain 1-day post fertilization (dpf) (Fig. 7D). While the fluorescence in the head region subsided after 1 dpf, its intensity in the posterior CNS region persisted through later stages, correlating with the reported *gfap* expression pattern (https://zfin.org) (Fig. S3). These results demonstrate that TICIT can be applied to generating transgenic animals in which an endogenous promoter of interest controls transgene expression.

## DISCUSSION

Developing research tools can serve as a potent catalyst for biomedical research. In recent years, CRISPR technologies have been widely embraced by the scientific community and have spawned tens of thousands of publications and applications (Anzalone et al., 2020). However, even with current CRISPR methods, the efficiencies for targeted integration of large DNA fragments in mammalian cells and various model organisms still need to improve, primarily due to their dependencies on cellular DNA repair mechanisms (Prill and Dawson, 2020; Yang et al., 2020). Hence, these methods are unsuitable for large-scale experimentation. In contrast, phiC31 integrase mediates DNA integration independently and with precision and high efficiency (Mosimann et al., 2013; Roberts et al., 2014). However, its recognition sites are not programmable. This study describes a two-step protocol for facile targeted integration of long DNA fragments into the zebrafish genome. A workflow of this method is provided in Fig. S4. As illustrated in Fig. 1, we show that this new technique, named TICIT, can open doors to diverse downstream applications for zebrafish research.

In the first step of this protocol, we successfully engineered a phiC31 landing pad in four genes via SpCas9 and ssODNs at a founder frequency ranging between 6-33%. This method is simple and effective and does not require DNA cloning. Moreover, using this method, researchers can place the attP site precisely to create or avoid an in-frame stop codon for different applications, as shown in this report.

In the second step of this protocol, phiC31 integrase is employed to insert an entire plasmid DNA at the target site. We only needed to screen a small number of fish to identify a founder. The founder frequency ranged between 16-100% at two sites – *attP^tyr_1^* and *attP^gfap^*. We showed that zebrafish lines carrying a functional landing site can be used repeatedly to generate multiple integration lines efficiently. These results demonstrate the value of the TICIT protocol. Nonetheless, this method is not without its limit. In the study, we did not detect any phiC31-mediated recombination at the *attP^tyr_2^* site. A defective or inaccessible attP landing site has been reported before (Mosimann et al., 2013). It is presently unknown why some attP landing sites are refractory to phiC31 integrase. Since there is no CpG dinucleotide that could be methylated in the attP sequence, it could be other DNA modifications or DNA-binding proteins that render some loci inaccessible to the enzyme. Variability in the efficiency of Cre-mediated recombination at different genomic locations has also been documented (Lalonde et al., 2022). Similarly, the phiC31 landing pad inserted in different genomic locations may have different integration efficiencies (Mosimann et al., 2013; Roberts et al., 2014). This should not preclude phiC31 integrase from being a powerful tool.

With this method, we developed an approach for marking zebrafish with fluorescent reporters indicating whether a fish is wild-type, heterozygous, or homozygous for an allele of interest. This technique allows genotyping by visual inspection and can save time and money for routine zebrafish work. It could be equally useful for researchers working with mice, rats, or other organisms. The technique may also enable new experiments by early identifying mutant animals from a mixed population. For example, it can be used to sort a homogeneous population of homozygous mutants for use in high-throughput screening for potential treatments of genetic disorders. We used the *ubi* promoter to enable early detection and easy sorting. It is possible that other promoters may be used.

Furthermore, the results showed that phiC31 mediated unidirectional integration with high fidelity since we could identify correct attR and attL sequences flanking the inserted DNA. This enables efficient in-frame insertion of a fluorescent marker into a targeted gene, which may be helpful when studying previously uncharacterized genes. An important consideration in any promoter-tagging design is the strength of the promoter of interest. For weaker promoters, a signal amplification strategy such as the UAS-Gal4 system may be needed (Wierson et al., 2020). In addition to reporter genes, researchers can also use TICIT to express other transgenes from an endogenous promoter. For example, by expressing the cDNAs of various genetic variants, this technique could be used for gene replacement in comparative studies of genetic variations.

One of the most significant benefits of single-copy, site-specific transgenesis compared to transgenesis via Tol2 transposon is the ability to obtain faithful transgene expression controlled by its promoter without positional and copy number artifacts. This could be achieved using phiC31 integrase and a zebrafish line carrying a ‘safe harbor’ or well-defined phiC31 docking locus. Hence, developing this technique may widen the use of the zebrafish as a platform to rapidly assess the functions of genetic variants in the coding and non-coding regions identified in genome-wide association studies (GWAS) (Edwards et al., 2013; Gusev et al., 2018; Tucker et al., 2017). Indeed, the phiC31 transgenesis method via several Tol2-generated attP lines has been shown to be superior to the Tol2 transgenesis method when evaluating human cis-regulatory elements in zebrafish (Bhatia et al., 2021; Roberts et al., 2014). The landing sites in the integration recipient lines used in previous studies have been mapped to intergenic and intragenic regions (Bhatia et al., 2021; Mosimann et al., 2013; Roberts et al., 2014). Here, we demonstrate that the *attP^tyr_1^* landing site exhibits high recombination efficiency. Fish harboring *ubi:EGFP* and *ubi:mCherry* in the *attP^tyr_1^* locus showed strong, broad, and consistent expression levels over several generations. It should be noted these data cannot preclude the *tyr* locus may confer genetic interactions with the *ubi* promoter or other regulatory elements inserted into this locus. In addition to the *ubi* promoter, investigation of the expression patterns of additional promoters in this locus is warranted. Though transgene integration at the *attP^tyr_1^* site will be accompanied by a single-copy deletion of the *tyr* gene, we have not observed any noticeable phenotypes in heterozygous *tyr* zebrafish, suggesting that the *attP^tyr_1^* fish may be used as transgenesis recipients for other purposes. Moreover, it would be interesting to explore more integration sites for phiC31 using CRISPR-Cas9.

We show that CRISPR-Cas9 and phiC31 technologies can be efficiently combined to construct novel genome engineering tools and zebrafish models. Presumably, it may be possible to perform attP insertion and DNA integration via phiC31 in one single step, which has been successfully demonstrated in human cells using the prime editor system instead of SpCas9 (Anzalone et al., 2022; Yarnall et al., 2023). Thus, this will be a useful future direction for zebrafish. Moreover, identifying other microbial DNA integrases that exhibit high efficiencies in zebrafish will be helpful, which may enable more sophisticated experimental designs involving multiple DNA integrations or cassette exchange (Durrant et al., 2023; Low et al., 2022; Yarnall et al., 2023). It can be expected that a broader adoption and more creative uses of the CRISPR and integrase technologies in zebrafish and other model organisms will play an important role in accelerating more transformative biomedical research in the near future.

## MATERIALS AND METHODS

### Generation of gRNAs and Cas9 protein

All gRNAs used in this study were produced via *in vitro* transcription as previously described (Gagnon et al., 2014). Target site and oligonucleotide sequences are listed in Tables S1 and S2, respectively. Briefly, a gene-specific oligonucleotide was annealed to a constant oligonucleotide encoding the CRISPR-Cas9 scaffold and then a fill-in reaction was performed using T4 DNA polymerase (New England Biolabs). For the *tyr_1* and *tyr_2* sites, the 76-nt SpCas9 (C9) scaffold was used. For gRNAs targeting *gfap* and *kcnh6a*, the 86-nt, enhanced SpCas9 (C9E) constant oligonucleotide was used. (Petri et al., 2022) The products were purified using Monarch^®^ PCR & DNA Cleanup Kit (New England Biolabs), which were then used as the template for *in vitro* transcription. *In vitro* transcription was performed using HiScribe™ T7 or SP6 High Yield RNA Synthesis Kit (New England Biolabs). RNA was subsequently purified using Monarch^®^ RNA Cleanup Kit (New England Biolabs).

For the generation of Cas9 protein, the plasmid pET-28b-Cas9-His (Addgene, #47327) was transformed into Rosetta (DE3) competent cells (Novagen) following the manufacturer's instructions. The production and purification of Cas9 protein were carried out as previously described (Gagnon et al., 2014). Single-use aliquots were stored at −80°C.

### Plasmid construction

To generate the plasmid for *in vitro* transcription of the mouse codon-optimized phiC31 integrase (phiC31o) mRNA, a T7 promoter was added to the pPhiC31o plasmid (Addgene, #13794). Briefly, two oligonucleotides, EcoRI-T7 and T7-EcoRI (Table S3), were hybridized with each other and ligated to EcoRI-linearized pPhiC31o. The construction (pT7_ PhiC31o) was transformed into NEB^®^ Turbo competent *E. coli* cells (New England Biolabs) and verified by Sanger sequencing after plasmid DNA extraction.

To generate pDestattB_ubi:mCherry, the mCherry coding sequence was PCR amplified from the plasmid pGFP-bait-MCS-NTR-mCherry using GoTaq^®^ DNA Polymerase (Promega) with primers listed in Table S3. PCR products were purified using the Monarch^®^ PCR & DNA Cleanup Kit (New England Biolabs) and then digested by BspHI and MfeI-HF (New England Biolabs). Meanwhile, the plasmid pDestattB_ubi:EGFP (Addgene, #68339) was digested by NcoI-HF and MfeI-HF (New England Biolabs) to generate the vector backbone. The digested mCherry and vector backbone fragments were purified using Zymoclean Gel DNA Recovery Kit (Zymo Research) and then ligated using the T4 DNA ligase (New England Biolabs). The construction was transformed into NEB^®^ Turbo competent *E. coli* cells (New England Biolabs) and verified by Sanger sequencing after plasmid DNA extraction.

To generate the pGEM-T_attB-P2A-EGFP plasmid, the EGFP coding sequence was PCR amplified from the plasmid pDestattB_ubi:EGFP using GoTaq^®^ DNA Polymerase (Promega) with attB-P2A-EGFP-F and pA-r primers (Table S3). PCR product was purified and ligated to the pGEM^®^-T vector (Promega). The construction was transformed into NEB^®^ Turbo competent *E. coli* cells (New England Biolabs) and a partial truncation in the resulting clone was discovered by Sanger sequencing in the attB-P2A-EGFP-F primer region. Hence, we designed another primer attB-P2A-EGFP-F1 (Table S3). The correct attB-P2A-EGFP sequence was PCR amplified from the plasmid with the truncated sequence using the attB-P2A-EGFP-F1 and pA-r primers. PCR product was purified and subcloned into pGEM^®^-T. The final construct pGEM-T_attB-P2A-EGFP was verified by Sanger sequencing.

### Oligonucleotide and mRNA synthesis

All oligonucleotides, including the ssODNs for attP knock-in, were ordered from Integrated DNA Technologies. The phiC31 integrase mRNA was prepared by *in vitro* transcription using *EcoRI*-linearized plasmid pCDNA3.1_phiC31 (Addgene, #68310) or *HindIII*-linearized plasmid pT7_PhiC31o as the template and mMESSAGE mMACHINE™ T7 Transcription Kit (Invitrogen). The former vector contains a phiC31 coding sequence (phiC31) (Bischof et al., 2007; Mosimann et al., 2013), whereas the latter contains a mouse codon-optimized phiC31 coding sequence (phiC31o) (Raymond and Soriano, 2007). RNA was subsequently purified using Monarch^®^ RNA Cleanup Kit (New England Biolabs).

### Zebrafish microinjection

All zebrafish husbandry and experiments were approved by the Massachusetts General Hospital Subcommittee on Research Animal Care and performed in accordance with the guidelines of the Institutional Animal Care and Use Committee at the Massachusetts General Hospital.

Microinjections were performed using the 1-cell stage of TuAB zebrafish embryos and approximately 2 nl of injection solution per embryo. For attP knock-in experiments, the injection solution contained 480 ng/µl of Cas9 protein, 230 ng/µl of gRNA, and 0.5-1 µM of ssODN. To prepare the injection mix, Cas9 protein and gRNA were combined and put at room temperature for 5 min before the ssODN was added. We later used 288 ng/µl of Cas9 protein, 70-80 ng/µl of gRNA, and 0.5 µM of ssODN for the *gfap* and *kcnh6a* target sites to circumvent embryo death and deformity caused by high rates of *gfap* and *kcnh6a* mutations. For plasmid DNA integration experiments, the injection solution contained 12.5 ng/µl of the phiC31 or 5 ng/µl of phiC31o mRNA and 12.5 ng/µl or 25 ng/µl of plasmid DNA (pDestattB_ubi:EGFP for the *tyr_1* and *tyr_2* targeted sites and pGEM-T-attB-P2A-EGFP for the *gfap* targeted site). We have used the mRNA of both phiC31 and phiC31o in these experiments and observed no consistent differences in their performance. For testing *gfap* and *kcnh6a* gRNA efficiencies, the injection solution contained 288 ng/µl of Cas9 protein and 80 ng/µl of gRNA. Injected embryos were incubated at 28.5°C after injection.

### Zebrafish genomic DNA extraction

Genomic DNA was extracted from fin clips of adult fish or embryos at 1 or 2 dpf. Zebrafish embryos that developed normally were lysed singly or as pools in lysis buffer (5 µl per embryo at 1 dpf, 8-10 µl per embryo at 2 dpf, and 30 µl per fin clip). The lysis buffer consisted of 10 mM Tris-HCl (pH 8.0), 2 mM EDTA (pH 8.0), 0.2% Triton X-100, and 0.5% Proteinase K. Lysates were incubated at 50°C overnight with occasional mixing till they turned clear, which were then heated at 95°C for 10 min to inactive Proteinase K. Genomic DNA was stored at 4°C.

### PCR-fluorescent fragment length (PCR-FFL) analysis and next-generation sequencing (NGS)

PCR-FFL analysis was employed to determine gRNA efficiencies or the sizes of the knock-in alleles (Foley et al., 2009). To prepare the samples, two-step PCR reactions were performed. In the first step, gene-specific forward and reverse primers were used to amplify the targeted loci, and the forward primers contained an 18-bp M13 sequence (5′- TGTAAAACGACGGCCAGT) at the 5′ end. The PCR product was diluted 100-fold and used for the second PCR reaction using the 5′ 6-FAM-labelled M13 forward primer and a gene-specific reverse primer. PCR primer sequences are listed in Table S3. The final products were analyzed at the Massachusetts General Hospital DNA Core.

For NGS, PCR amplicons (generally less than 280 bps) encompassing the targeted loci were generated using 1 µl of the zebrafish lysate with Phusion^®^ High-Fidelity Polymerase (New England Biolabs) and primers listed in Table S3. PCR products were purified using the Monarch^®^ PCR & DNA Cleanup Kit (New England Biolabs) and submitted to the Massachusetts General Hospital DNA Core. Sequencing data were analyzed with CRISResso2 using the HDR mode (http://crispresso2.pinellolab.org/submission).

### Zebrafish genotyping, line generation, and founder screens

The sequences of all PCR primers for genotyping are listed in Table S3. To generate attP knock-in lines, embryos microinjected with SpCas9, gRNA, and attP knock-in ssODN were raised to maturity and screened for founders. Potential founders (F_0_) were outcrossed to the wild-type fish, and their progeny (F_1_) were genotyped in pools (five embryos per pool) via two-step nested PCR. The first PCR was to amplify the targeted loci, and the second PCR was to detect the attP insertion at the targeted loci (Table S3). Alternatively, when F_1_ progeny were lysed individually, only the second step of PCR was needed to detect the attP insertion. Once an attP insertion was detected by PCR, the knock-in alleles were further verified by Sanger sequencing or next-generation sequencing, all using gene-specific primers to amplify the targeted loci (Table S3). For sequence confirmation by Sanger sequencing, PCR products were subcloned using the pGEM-T vector, followed by colony PCR to identify the clones containing the desired allele. Subsequently, the plasmid DNA was extracted and submitted to the Massachusetts General Hospital DNA Core for sequencing. For genotyping of F_1_ and F_2_ adult zebrafish, fish were anesthetized briefly with tricaine, and a small fin biopsy was taken for DNA extraction as described above. Gene-specific primers were used to amplify the targeted loci (Table S3), and the samples containing the knock-in alleles should yield PCR products corresponding to both the wild-type allele and the knock-in allele. For further verification, Sanger sequencing was employed.

To generate allele-tracking reporter lines, embryos from heterozygous *attP^tyr_1^* fish incrosses or outcrosses with the wild-type fish were injected with the phiC31 mRNA together with pDestattB_ubi:EGFP or pDestattB_ubi:mCherry DNA. The injected embryos were raised to adulthood and screened for founders. The progeny of potential founders was first screened for fluorescence. Fluorescent F_1_ embryos were lysed singly, and PCR was performed to detect DNA integration at the targeted locus (Table S3). Sanger sequencing was used to confirm the sequences of attL and attR at the junctions of the integrated DNA. Subsequently, fluorescent progeny (F_1_) from the confirmed founders were raised to adulthood and genotyped by fin clipping and PCR as described above. We used two criteria to determine that a heterozygous *ubi:EGFP^tyr^* F_1_ fish did not carry a second integration outside of the targeted locus. First, when a heterozygous fish was outcrossed to a wild-type fish, it should produce approximately 50% of the fluorescent progeny. Second, all fluorescent F_2_ progeny should harbor the correct integration. F_1_ fish that fit these criteria were used to propagate all future generations.

To generate a target gene-specific reporter line, embryos from heterozygous *attP^gfap^* fish outcrossed to wild-type fish were injected with the phiC31 mRNA together with pGEM-T_attB-P2A-EGFP DNA. The injected embryos were raised to adulthood and screened for founders. The founder screen was performed as described for creating allele-tracking reporter lines.

### Confocal imaging of the gfap:gfp transgenic embryos

Heterozygous *P2A-EGFP^gfap^* embryos were obtained by mating heterozygous *P2A-EGFP^gfap^* fish with wild-type TuAB fish. EGFP-positive embryos were selected for imaging at 1- and 2-dpf. Embryos were treated with 0.03 g/l phenylthiourea (PTU) to inhibit pigmentation, and manually dechorionated using tweezers. Right before imaging, embryos were anesthetized by 30 mg/l tricaine-S (Western Chemical) and mounted in 2% low melting agarose (LONZA) for dorsal or lateral view in 35 mm Petri dishes with a glass bottom. Imaging was performed on ZEISS LSM900 confocal microscope using a 10X objective. Z-stacks were collected with an interval of 3 µm. Images were stitched and computed as the sum of projections in ImageJ.

### HCR™ RNA-FISH staining

HCR™ reagents including probe sets, amplifiers, and buffers, were purchased from Molecular Instruments (Los Angeles, CA, USA). The probe set was designed for Zebrafish *gfap* (NM_131373.2) and the *EGFP* mRNA sequences. Each probe set is composed of multiple probe pairs hybridized to different regions along the target mRNA. The HCR amplifiers for the *gfap* and *EGFP* mRNA targets were fluorescently labeled with AlexaFluor 546 and AlexaFluor 488, respectively. The staining procedure followed a modified version of the ‘HCR RNA-FISH protocol for whole-mount zebrafish embryos and larvae’ provided by the manufacturer. Briefly, embryos were anesthetized by immersing them in ice water and fixed with 4% paraformaldehyde (PFA) in phosphate-buffered saline for 1 h at 4°C. The samples were then permeabilized with proteinase K (final concentration 15 μg/ml) for 20 min, post-fixed in 4% PFA, and incubated overnight at 37°C in probe hybridization buffer containing 4 pmol of each probe (final concentration 16 nM). The probes were washed at 37°C with wash buffer before overnight incubation in amplification buffer containing 30 pmol of each fluorescently labeled amplifier at room temperature in a dark drawer. Embryos were then washed three times with 5X SSCT (0.75 M NaCl, 75 mM sodium citrate, 0.1% Tween-20). Finally, the embryos were ready for imaging.

To take images, embryos were mounted to glass-bottom dishes in 1% low-melting agarose gel in 1X E3 and imaged with a ZEIS LSM700 confocal scanning microscope using either 5X or 10X magnification. Images were obtained both with single z-planes and z-stacks. Imaging was performed with the following lasers: Diode 555 and Diode 488 for detecting the *gfap* and *EGFP* mRNA, respectively. Any post-capture image processing was performed uniformly to wild-type and transgenic fish embryos.

### Histology and cytology

Adult zebrafish were euthanized using approved protocols with tricaine and then fixed in 4% PFA. Paraffin embedding, sectioning, H&E staining, and immunohistochemistry (IHC) for EGFP were performed using standard protocols. EGFP was detected using the JL-8 mouse monoclonal antibody (Clontech). For IHC, the primary antibody was diluted at 1:200, and the secondary antibody, an HRP-conjugated, donkey anti-rabbit antibody (Biovision), was used at a dilution of 1:1000.

For flow cytometry analysis of hematopoietic cells, *ubi:EGFP^tyr^* adult zebrafish were euthanized prior to kidney collection. The kidney was dissected and placed into ice-cold 0.9X phosphate-buffered saline (PBS) containing 5% fetal bovine serum. Whole kidney marrow (WKM) cells in single-cell suspension were generated by gently triturating and by passing through a 40-μm filter. FACS was conducted on CytoFLEX (Beckman Coulter, NJ, USA) and data were analyzed with FlowJo software (Tree Star, OR, USA). Various hematopoietic cell populations were identified as previously reported (Traver et al., 2003).

## Supplementary Material

10.1242/biolopen.061666_sup1 Supplementary information

## References

[BIO061666C1] Abe, G., Suster, M. L. and Kawakami, K. (2011). Tol2-mediated transgenesis, gene trapping, enhancer trapping, and the Gal4-UAS system. *Methods Cell Biol.* 104, 23-49. 10.1016/B978-0-12-374814-0.00002-1 21924155

[BIO061666C2] Anzalone, A. V., Koblan, L. W. and Liu, D. R. (2020). Genome editing with CRISPR-Cas nucleases, base editors, transposases and prime editors. *Nat. Biotechnol.* 38, 824-844. 10.1038/s41587-020-0561-9 32572269

[BIO061666C3] Anzalone, A. V., Gao, X. D., Podracky, C. J., Nelson, A. T., Koblan, L. W., Raguram, A., Levy, J. M., Mercer, J. A. M. and Liu, D. R. (2022). Programmable deletion, replacement, integration and inversion of large DNA sequences with twin prime editing. *Nat. Biotechnol.* 40, 731-740. 10.1038/s41587-021-01133-w 34887556 PMC9117393

[BIO061666C4] Auer, T. O., Duroure, K., De Cian, A., Concordet, J. P. and Del Bene, F. (2013). Highly efficient CRISPR/Cas9-mediated knock-in in zebrafish by homology-independent DNA repair. *Genome Res.* 24, 142-153. 10.1101/gr.161638.113 24179142 PMC3875856

[BIO061666C5] Belteki, G., Gertsenstein, M., Ow, D. W. and Nagy, A. (2003). Site-specific cassette exchange and germline transmission with mouse ES cells expressing phiC31 integrase. *Nat. Biotechnol.* 21, 321-324. 10.1038/nbt787 12563279

[BIO061666C6] Bhatia, S., Kleinjan, D. J., Uttley, K., Mann, A., Dellepiane, N. and Bickmore, W. A. (2021). Quantitative spatial and temporal assessment of regulatory element activity in zebrafish. *eLife* 10, e65601. 10.7554/eLife.65601 34796872 PMC8604437

[BIO061666C7] Bischof, J., Maeda, R. K., Hediger, M., Karch, F. and Basler, K. (2007). An optimized transgenesis system for Drosophila using germ-line-specific phiC31 integrases. *Proc. Natl. Acad. Sci. USA* 104, 3312-3317. 10.1073/pnas.0611511104 17360644 PMC1805588

[BIO061666C8] Boel, A., De Saffel, H., Steyaert, W., Callewaert, B., De Paepe, A., Coucke, P. J. and Willaert, A. (2018). CRISPR/Cas9-mediated homology-directed repair by ssODNs in zebrafish induces complex mutational patterns resulting from genomic integration of repair-template fragments. *Dis. Model. Mech.* 11, dmm035352. 10.1242/dmm.035352 30355591 PMC6215429

[BIO061666C9] Calos, M. P. (2006). The phiC31 integrase system for gene therapy. *Curr. Gene Ther.* 6, 633-645. 10.2174/156652306779010642 17168696

[BIO061666C10] Chen, C. M., Krohn, J., Bhattacharya, S. and Davies, B. (2011). A comparison of exogenous promoter activity at the ROSA26 locus using a PhiiC31 integrase mediated cassette exchange approach in mouse ES cells. *PLoS One* 6, e23376. 10.1371/journal.pone.0023376 21853122 PMC3154917

[BIO061666C11] Choi, T. Y., Choi, T. I., Lee, Y. R., Choe, S. K. and Kim, C. H. (2021). Zebrafish as an animal model for biomedical research. *Exp. Mol. Med.* 53, 310-317. 10.1038/s12276-021-00571-5 33649498 PMC8080808

[BIO061666C12] Cully, M. (2019). Zebrafish earn their drug discovery stripes. *Nat. Rev. Drug Discov.* 18, 811-813. 10.1038/d41573-019-00165-x 31673135

[BIO061666C13] Demarest, S. T. and Brooks-Kayal, A. (2018). From molecules to medicines: the dawn of targeted therapies for genetic epilepsies. *Nat. Rev. Neurol.* 14, 735-745. 10.1038/s41582-018-0099-3 30448857

[BIO061666C14] DiNapoli, S. E., Martinez-McFaline, R., Gribbin, C. K., Wrighton, P. J., Balgobin, C. A., Nelson, I., Leonard, A., Maskin, C. R., Shwartz, A., Quenzer, E. D. et al. (2020). Synthetic CRISPR/Cas9 reagents facilitate genome editing and homology directed repair. *Nucleic Acids Res.* 48, e38. 10.1093/nar/gkaa085 32064511 PMC7144937

[BIO061666C15] Durrant, M. G., Fanton, A., Tycko, J., Hinks, M., Chandrasekaran, S. S., Perry, N. T., Schaepe, J., Du, P. P., Lotfy, P., Bassik, M. C. et al. (2023). Systematic discovery of recombinases for efficient integration of large DNA sequences into the human genome. *Nat. Biotechnol.* 41, 488-499. 10.1038/s41587-022-01494-w 36217031 PMC10083194

[BIO061666C16] Edwards, S. L., Beesley, J., French, J. D. and Dunning, A. M. (2013). Beyond GWASs: illuminating the dark road from association to function. *Am. J. Hum. Genet.* 93, 779-797. 10.1016/j.ajhg.2013.10.012 24210251 PMC3824120

[BIO061666C17] Fazio, M., Ablain, J., Chuan, Y., Langenau, D. M. and Zon, L. I. (2020). Zebrafish patient avatars in cancer biology and precision cancer therapy. *Nat. Rev. Cancer* 20, 263-273. 10.1038/s41568-020-0252-3 32251397 PMC8011456

[BIO061666C18] Foley, J. E., Maeder, M. L., Pearlberg, J., Joung, J. K., Peterson, R. T. and Yeh, J. R. (2009). Targeted mutagenesis in zebrafish using customized zinc-finger nucleases. *Nat. Protoc.* 4, 1855-1867. 10.1038/nprot.2009.209 20010934 PMC2814337

[BIO061666C19] Gagnon, J. A., Valen, E., Thyme, S. B., Huang, P., Akhmetova, L., Pauli, A., Montague, T. G., Zimmerman, S., Richter, C. and Schier, A. F. (2014). Efficient mutagenesis by Cas9 protein-mediated oligonucleotide insertion and large-scale assessment of single-guide RNAs. *PLoS One* 9, e98186. 10.1371/journal.pone.0098186 24873830 PMC4038517

[BIO061666C20] Gusev, A., Mancuso, N., Won, H., Kousi, M., Finucane, H. K., Reshef, Y., Song, L., Safi, A., Schizophrenia Working Group of the Psychiatric Genomics Consortium, McCarroll, S. et al. (2018). Transcriptome-wide association study of schizophrenia and chromatin activity yields mechanistic disease insights. *Nat. Genet.* 50, 538-548. 10.1038/s41588-018-0092-1 29632383 PMC5942893

[BIO061666C21] Gut, P., Reischauer, S., Stainier, D. Y. R. and Arnaout, R. (2017). Little fish, big data: zebrafish as a model for cardiovascular and metabolic disease. *Physiol. Rev.* 97, 889-938. 10.1152/physrev.00038.2016 28468832 PMC5817164

[BIO061666C22] Helenius, I. T. and Yeh, J. R. (2012). Small zebrafish in a big chemical pond. *J. Cell. Biochem.* 113, 2208-2216. 10.1002/jcb.24120 22396148 PMC3349782

[BIO061666C23] Hillman, R. T. and Calos, M. P. (2012). Site-specific integration with bacteriophage PhiC31 integrase. *Cold Spring Harb Protoc* 2012, pdb.prot069211. 10.1101/pdb.prot069211 22550292

[BIO061666C24] Hisano, Y., Sakuma, T., Nakade, S., Ohga, R., Ota, S., Okamoto, H., Yamamoto, T. and Kawahara, A. (2015). Precise in-frame integration of exogenous DNA mediated by CRISPR/Cas9 system in zebrafish. *Sci. Rep.* 5, 8841. 10.1038/srep08841 25740433 PMC4350073

[BIO061666C25] Hoshijima, K., Jurynec, M. J., Klatt Shaw, D., Jacobi, A. M., Behlke, M. A. and Grunwald, D. J. (2019). Highly efficient CRISPR-Cas9-based methods for generating deletion mutations and f0 embryos that lack gene function in zebrafish. *Dev. Cell* 51, 645-657.e644. 10.1016/j.devcel.2019.10.004 31708433 PMC6891219

[BIO061666C26] Jao, L. E., Wente, S. R. and Chen, W. (2013). Efficient multiplex biallelic zebrafish genome editing using a CRISPR nuclease system. *Proc. Natl. Acad. Sci. USA* 110, 13904-13909. 10.1073/pnas.1308335110 23918387 PMC3752207

[BIO061666C27] Kirchmaier, S., Hockendorf, B., Moller, E. K., Bornhorst, D., Spitz, F. and Wittbrodt, J. (2013). Efficient site-specific transgenesis and enhancer activity tests in medaka using PhiC31 integrase. *Development* 140, 4287-4295. 10.1242/dev.096081 24048591 PMC3809364

[BIO061666C28] Kong, Q., Hai, T., Ma, J., Huang, T., Jiang, D., Xie, B., Wu, M., Wang, J., Song, Y., Wang, Y. et al. (2014). Rosa26 locus supports tissue-specific promoter driving transgene expression specifically in pig. *PLoS One* 9, e107945. 10.1371/journal.pone.0107945 25232950 PMC4169413

[BIO061666C29] Lalonde, R. L., Kemmler, C. L., Riemslagh, F. W., Aman, A. J., Kresoja-Rakic, J., Moran, H. R., Nieuwenhuize, S., Parichy, D. M., Burger, A. and Mosimann, C. (2022). Heterogeneity and genomic loci of ubiquitous transgenic Cre reporter lines in zebrafish. *Dev. Dyn.* 251, 1754-1773. 10.1002/dvdy.499 35582941 PMC10069295

[BIO061666C30] Li, Y. E., Allen, B. G. and Weeks, D. L. (2012). Using PhiC31 integrase to mediate insertion of DNA in Xenopus embryos. *Methods Mol. Biol.* 917, 219-230. 10.1007/978-1-61779-992-1_13 22956091 PMC3551469

[BIO061666C31] Low, B. E., Hosur, V., Lesbirel, S. and Wiles, M. V. (2022). Efficient targeted transgenesis of large donor DNA into multiple mouse genetic backgrounds using bacteriophage Bxb1 integrase. *Sci. Rep.* 12, 5424. 10.1038/s41598-022-09445-w 35361849 PMC8971409

[BIO061666C32] MacRae, C. A. and Peterson, R. T. (2015). Zebrafish as tools for drug discovery. *Nat. Rev. Drug Discov.* 14, 721-731. 10.1038/nrd4627 26361349

[BIO061666C33] Meyer, M., de Angelis, M. H., Wurst, W. and Kuhn, R. (2010). Gene targeting by homologous recombination in mouse zygotes mediated by zinc-finger nucleases. *Proc. Natl. Acad. Sci. USA* 107, 15022-15026. 10.1073/pnas.1009424107 20686113 PMC2930558

[BIO061666C34] Moreno-Mateos, M. A., Fernandez, J. P., Rouet, R., Vejnar, C. E., Lane, M. A., Mis, E., Khokha, M. K., Doudna, J. A. and Giraldez, A. J. (2017). CRISPR-Cpf1 mediates efficient homology-directed repair and temperature-controlled genome editing. *Nat. Commun.* 8, 2024. 10.1038/s41467-017-01836-2 29222508 PMC5722943

[BIO061666C35] Mosimann, C., Kaufman, C. K., Li, P., Pugach, E. K., Tamplin, O. J. and Zon, L. I. (2011). Ubiquitous transgene expression and Cre-based recombination driven by the ubiquitin promoter in zebrafish. *Development* 138, 169-177. 10.1242/dev.059345 21138979 PMC2998170

[BIO061666C36] Mosimann, C., Puller, A. C., Lawson, K. L., Tschopp, P., Amsterdam, A. and Zon, L. I. (2013). Site-directed zebrafish transgenesis into single landing sites with the phiC31 integrase system. *Dev. Dyn.* 242, 949-963. 10.1002/dvdy.23989 23723152 PMC3775328

[BIO061666C37] Patton, E. E., Zon, L. I. and Langenau, D. M. (2021). Zebrafish disease models in drug discovery: from preclinical modelling to clinical trials. *Nat. Rev. Drug Discov.* 20, 611-628. 10.1038/s41573-021-00210-8 34117457 PMC9210578

[BIO061666C38] Petri, K., Zhang, W., Ma, J., Schmidts, A., Lee, H., Horng, J. E., Kim, D. Y., Kurt, I. C., Clement, K., Hsu, J. Y. et al. (2022). CRISPR prime editing with ribonucleoprotein complexes in zebrafish and primary human cells. *Nat. Biotechnol.* 40, 189-193. 10.1038/s41587-021-00901-y 33927418 PMC8553808

[BIO061666C39] Prill, K. and Dawson, J. F. (2020). Homology-directed repair in zebrafish: witchcraft and wizardry? *Front Mol Biosci* 7, 595474. 10.3389/fmolb.2020.595474 33425990 PMC7793982

[BIO061666C40] Prykhozhij, S. V., Fuller, C., Steele, S. L., Veinotte, C. J., Razaghi, B., Robitaille, J. M., McMaster, C. R., Shlien, A., Malkin, D. and Berman, J. N. (2018). Optimized knock-in of point mutations in zebrafish using CRISPR/Cas9. *Nucleic Acids Res.* 46, e102. 10.1093/nar/gky512 29905858 PMC6158492

[BIO061666C41] Raymond, C. S. and Soriano, P. (2007). High-efficiency FLP and PhiC31 site-specific recombination in mammalian cells. *PLoS One* 2, e162. 10.1371/journal.pone.0000162 17225864 PMC1764711

[BIO061666C42] Richardson, C. D., Ray, G. J., DeWitt, M. A., Curie, G. L. and Corn, J. E. (2016). Enhancing homology-directed genome editing by catalytically active and inactive CRISPR-Cas9 using asymmetric donor DNA. *Nat. Biotechnol.* 34, 339-344. 10.1038/nbt.3481 26789497

[BIO061666C43] Rissone, A. and Burgess, S. M. (2018). Rare genetic blood disease modeling in zebrafish. *Front. Genet.* 9, 348. 10.3389/fgene.2018.00348 30233640 PMC6127601

[BIO061666C44] Roberts, J. A., Miguel-Escalada, I., Slovik, K. J., Walsh, K. T., Hadzhiev, Y., Sanges, R., Stupka, E., Marsh, E. K., Balciuniene, J., Balciunas, D. et al. (2014). Targeted transgene integration overcomes variability of position effects in zebrafish. *Development* 141, 715-724. 10.1242/dev.100347 24449846 PMC3899822

[BIO061666C45] Sakai, C., Ijaz, S. and Hoffman, E. J. (2018). Zebrafish models of neurodevelopmental disorders: past, present, and future. *Front. Mol. Neurosci.* 11, 294. 10.3389/fnmol.2018.00294 30210288 PMC6123572

[BIO061666C46] Shin, J., Chen, J. and Solnica-Krezel, L. (2014). Efficient homologous recombination-mediated genome engineering in zebrafish using TALE nucleases. *Development* 141, 3807-3818. 10.1242/dev.108019 25249466 PMC4197590

[BIO061666C47] Soriano, P. (1999). Generalized lacZ expression with the ROSA26 Cre reporter strain. *Nat. Genet.* 21, 70-71. 10.1038/5007 9916792

[BIO061666C48] Swinney, D. C. and Anthony, J. (2011). How were new medicines discovered? *Nat. Rev. Drug Discov.* 10, 507-519. 10.1038/nrd3480 21701501

[BIO061666C49] Thorpe, H. M. and Smith, M. C. (1998). In vitro site-specific integration of bacteriophage DNA catalyzed by a recombinase of the resolvase/invertase family. *Proc. Natl. Acad. Sci. USA* 95, 5505-5510. 10.1073/pnas.95.10.5505 9576912 PMC20407

[BIO061666C50] Torraca, V. and Mostowy, S. (2018). Zebrafish Infection: From Pathogenesis to Cell Biology. *Trends Cell Biol.* 28, 143-156. 10.1016/j.tcb.2017.10.002 29173800 PMC5777827

[BIO061666C51] Traver, D., Paw, B. H., Poss, K. D., Penberthy, W. T., Lin, S. and Zon, L. I. (2003). Transplantation and in vivo imaging of multilineage engraftment in zebrafish bloodless mutants. *Nat. Immunol.* 4, 1238-1246. 10.1038/ni1007 14608381

[BIO061666C52] Tucker, N. R., Dolmatova, E. V., Lin, H., Cooper, R. R., Ye, J., Hucker, W. J., Jameson, H. S., Parsons, V. A., Weng, L. C., Mills, R. W. et al. (2017). Diminished PRRX1 expression is associated with increased risk of atrial fibrillation and shortening of the cardiac action potential. *Circ. Cardiovasc. Genet.* 10, e001902. 28974514 10.1161/CIRCGENETICS.117.001902 PMC5679717

[BIO061666C53] Wierson, W. A., Welker, J. M., Almeida, M. P., Mann, C. M., Webster, D. A., Torrie, M. E., Weiss, T. J., Kambakam, S., Vollbrecht, M. K., Lan, M. et al. (2020). Efficient targeted integration directed by short homology in zebrafish and mammalian cells. *eLife* 9, e53968. 10.7554/eLife.53968 32412410 PMC7228771

[BIO061666C54] Yang, H., Ren, S., Yu, S., Pan, H., Li, T., Ge, S., Zhang, J. and Xia, N. (2020). Methods favoring homology-directed repair choice in response to CRISPR/Cas9 induced-double strand breaks. *Int. J. Mol. Sci.* 21, 6461. 10.3390/ijms21186461 32899704 PMC7555059

[BIO061666C55] Yarnall, M. T. N., Ioannidi, E. I., Schmitt-Ulms, C., Krajeski, R. N., Lim, J., Villiger, L., Zhou, W., Jiang, K., Garushyants, S. K., Roberts, N. et al. (2023). Drag-and-drop genome insertion of large sequences without double-strand DNA cleavage using CRISPR-directed integrases. *Nat. Biotechnol.* 41, 500-512. 10.1038/s41587-022-01527-4 36424489 PMC10257351

[BIO061666C56] Zhang, Y., Huang, H., Zhang, B. and Lin, S. (2016). TALEN- and CRISPR-enhanced DNA homologous recombination for gene editing in zebrafish. *Methods Cell Biol.* 135, 107-120. 10.1016/bs.mcb.2016.03.005 27443922

