# Supplementary material for: Instantaneous visual genotyping and facile site-specific transgenesis via CRISPR-Cas9 and phiC31 integrase
Source: Biol Open. 2024 Sep 3;13(9):bio061666. doi: 10.1242/bio.061666 (PMC11391820; doi:10.1242/bio.061666)
Supplement: Supplementary information [file biolopen-13-061666-s1.pdf]

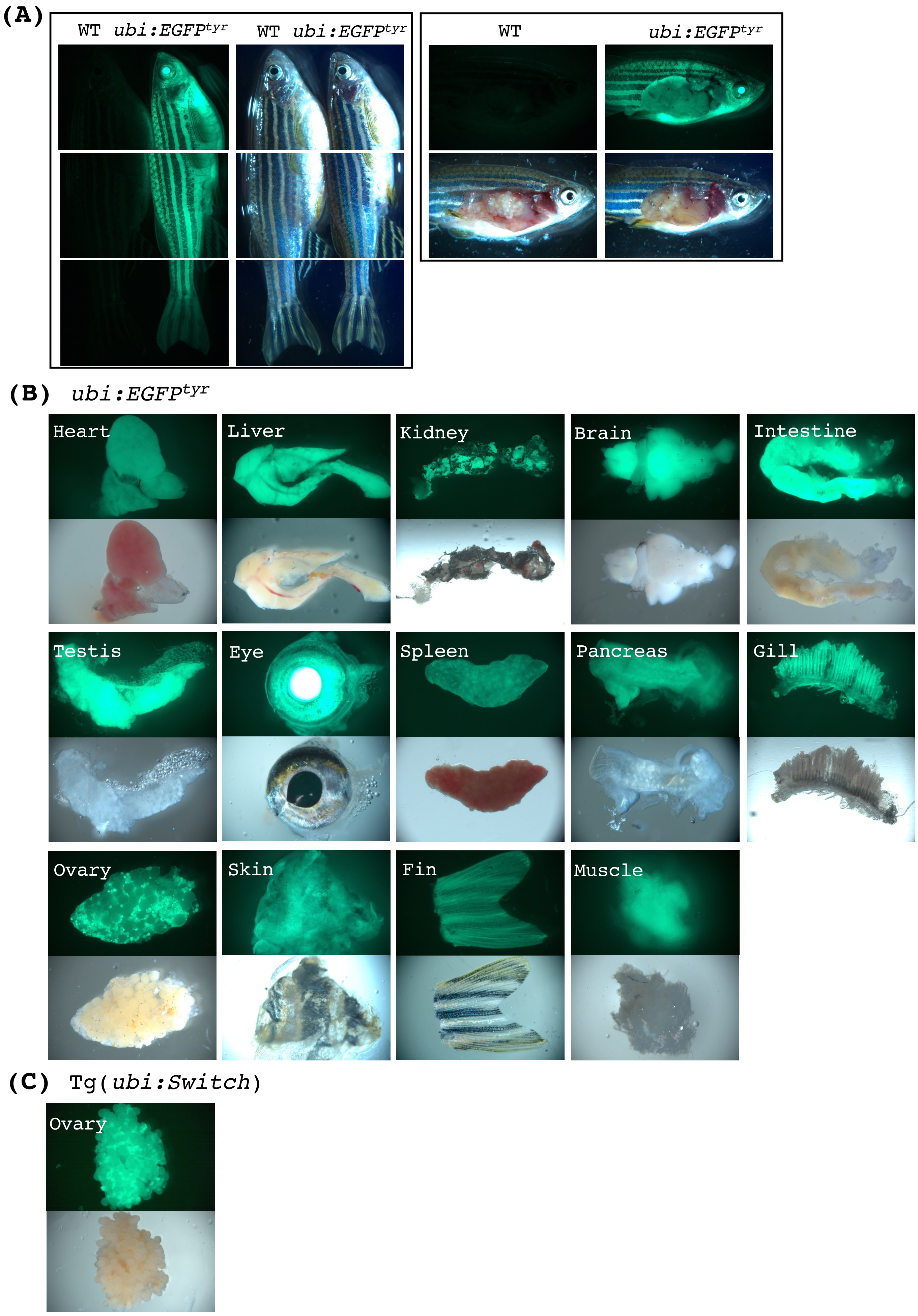

Fig. S1. EGFP fluorescence can be seen in multiple organs and tissues of *ubi:EGFP^{tyr}* adult fish.

(A) Bright-field and fluorescence images of *ubi:EGFP^{tyr}* and wild-type (WT) adult fish. Broad EGFP expression are detected in *ubi:EGFP^{tyr}* fish, though fluorescence may be obscured by pigmentation in some regions.

(B) Bright-field and fluorescence images of various organs and tissues isolated from *ubi:EGFP^{tyr}* adult fish. Broad EGFP expression patterns are detected. Note the fluorescence in the ovary indicates that EGFP expression can be detected in immature oocytes but not in mature oocytes.

(C) Bright-field and fluorescence images of the ovary from Tg(*ubi:Switch)* adult female fish. Note that EGFP expression can be detected in both immature and mature oocytes.

Biology Open • Supplementary information

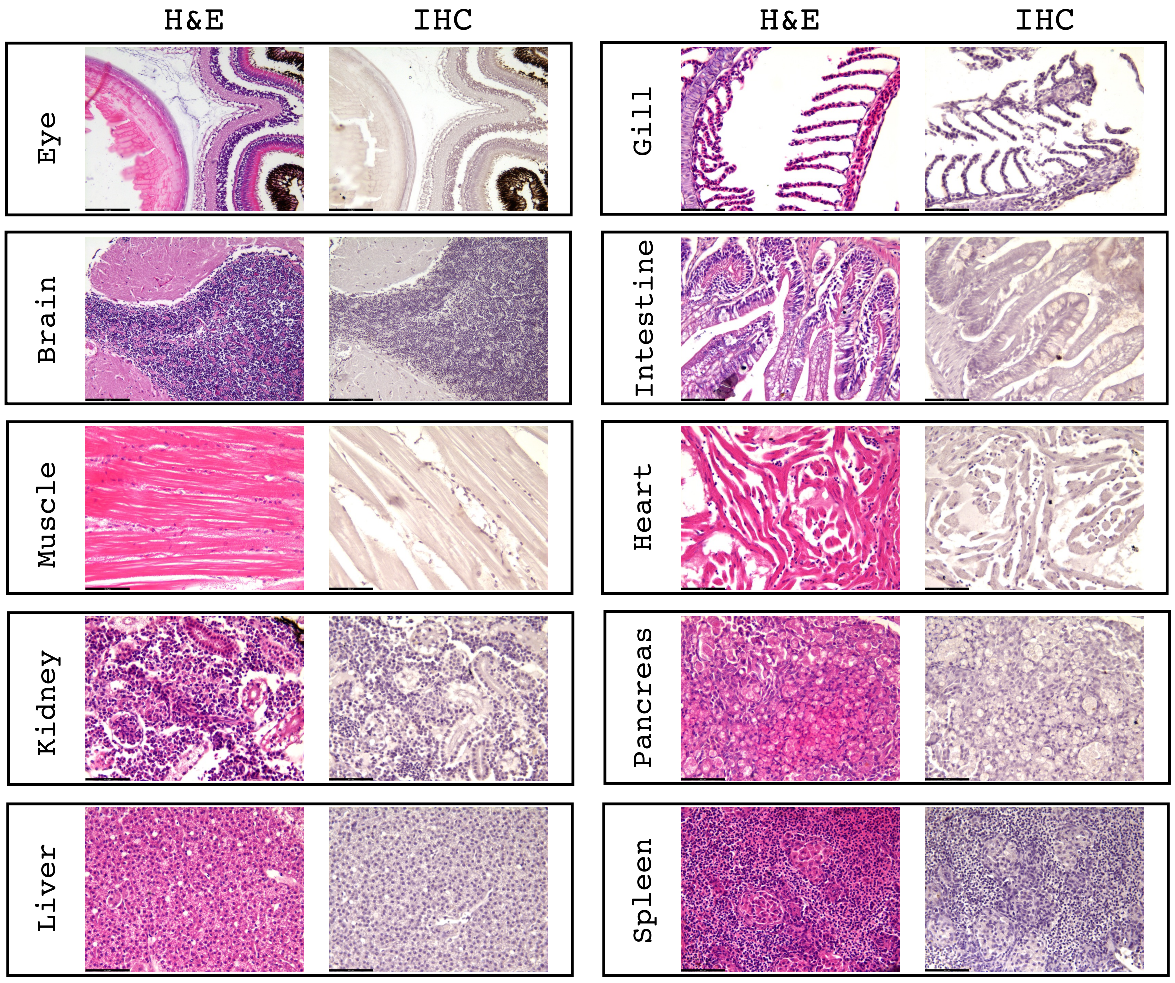

Fig. S2. GFP immunohistochemistry (IHC) staining of wild-type adult fish. Histology sections of wild-type adult fish stained with H&E or anti-EGFP antibody (IHC). These samples were harvested, processed, stained, and imaged simultaneously with the samples shown in **Figure 6**. All images were acquired under 400X magnification.

Biology Open • Supplementary information

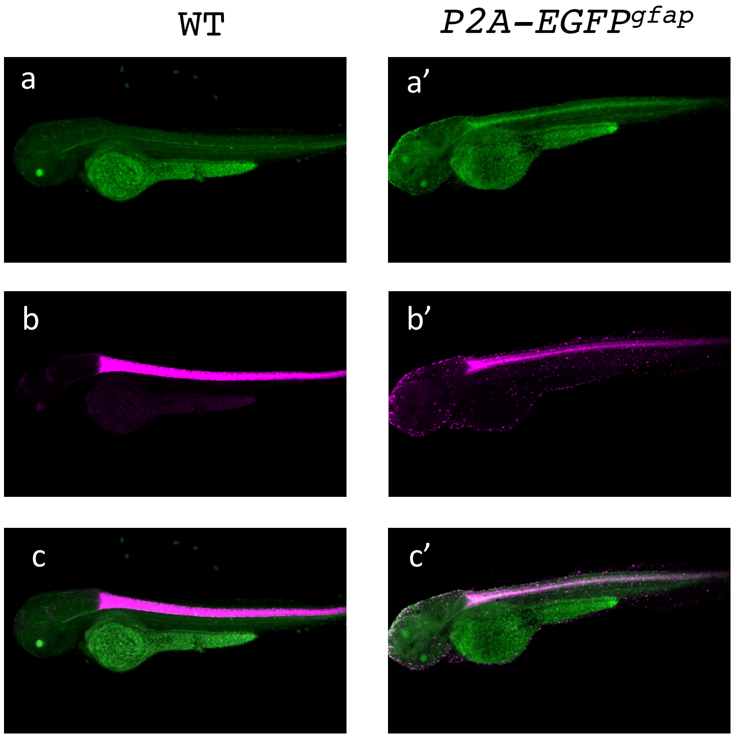

Fig. S3. Colocalization of endogenous *gfap* mRNA and EGFP reporter mRNA in Tg(P2A-EGFP^{gfap}) and wild-type embryos at 2 dpf. In situ RNA hybridization chain reaction (HCR) was used to detect the colocalization of *gfap* (magenta) and EGFP (green) mRNA in the heterozygous Tg(P2A-EGFP^{gfap}) embryos at 2 dpf. Strong EGFP expression was observed in the spinal cord of Tg(P2A-EGFP^{gfap}) embryos (a'), while both wild-type and Tg(P2A-EGFP^{gfap}) embryos showed autofluorescence and non-specific background staining in the lens and yolk (a, a'). Endogenous *gfap* mRNA was detected in the spinal cord of both wild-type and Tg(P2A-EGFP^{gfap}) embryos (b, b'). The merged image of the wild-type embryo shows no colocalization due to lack of EGFP expression (c), while the merged image of Tg(P2A-EGFP^{gfap}) embryo demonstrates colocalization (white) between *gfap* and EGFP in the spinal cord (c').

Biology Open • Supplementary information

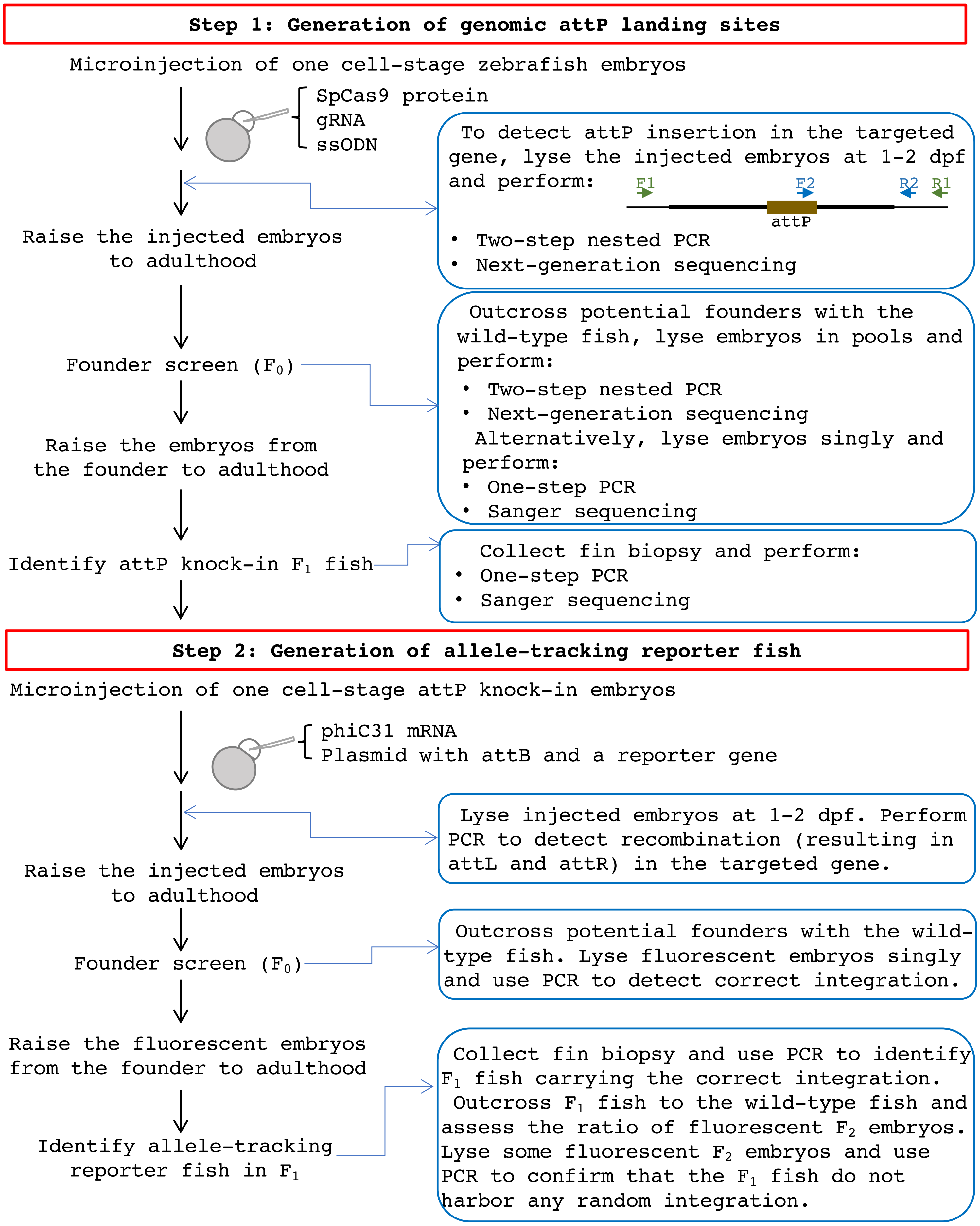

Fig. S4. The workflow for the generation of allele-tracking reporter zebrafish using TICIT.

The flowchart outlines the procedures for generating allele-tracking reporter zebrafish.

Biology Open • Supplementary information

Table S1. SpCas9 target sites tested or used in this study. In the 'Target site sequence', PAM sequence is underlined. In the 'gRNA spacer sequence', an extra 'G' (shown in red) is added to faciliate efficient in vitro transcription. For attP knock-in, kcnh6a_2 and gfap_1 gRNAs were used.

Gene	gRNA Name	Target site sequence (5'->3')	Exon	Direction	gRNA spacer sequence (5' -> 3')
tyr	tyr_1	GGACTGGAGGACTTCTGGGGAGG	1	forward	CCCCAGAAGTCCTCCAGTCC
tyr	tyr_2	GGGCCGCAGTATCCTCACTCAGG	1	reverse	GGGCCGCAGTATCCTCACTC
kcnh6a	kcnh6a_1	AGCGACATGTCCGCGGCGCACGG	1	Reverse	GGCGACATGTCCGCGGCGCA
kcnh6a	kcnh6a_2	GAAAAATGCCCGTGCGCCGCGG	1	forward	GAAAAATGCCCGTGCGCCG
gfap	gfap_1	GGAACGCTGGGACTCCATGGTGG	1	reverse	GGAACGCTGGGACTCCATGG
gfap	gfap_2	GAAGGAACGCTGGGACTCCATGG	1	reverse	GAAGGAACGCTGGGACTCCA
gfap	gfap_3	GGTGCCGAATCTCTTTCGGTAGG	1	reverse	GGTGCCGAATCTCTTTCGGT

Table S2. Oligonucleotides used for gRNA construction. In 'Sequnce', T7 or SP6 promoter sequences are shown in green, and the sequences complementary to the C9 or C9E constant oligo are shown in blue.

Oligo name	Sequence 5'-> 3'	Note
C9E_tyr_1	TAATACGACTCACTATACCCCAGAAGTCCTCCAGTCCGTTTTAGAGCTAGAA	Gene-specific forward primer
C9E_tyr_2	TAATACGACTCACTATAGGGCCGCAGTATCCTCACTCGTTTTAGAGCTAGAA	Gene-specific forward primer
C9E_zKcnh6a_1	ATTTAGGTGACACTATAGGCGACATGTCCGCGGCGCAGTTTAAGAGCTATGCTGGAAACA	Gene-specific forward primer
C9E_zKcnh6a_2	ATTTAGGTGACACTATAGAAAAATGCCCGTGCGCCGGTTTAAGAGCTATGCTGGAAACA	Gene-specific forward primer
C9E_zGfap_1	ATTTAGGTGACACTATAGGAACGCTGGGACTCCATGGGTTTAAGAGCTATGCTGGAAACA	Gene-specific forward primer
C9E_zGfap_2	ATTTAGGTGACACTATAGAAGGAACGCTGGGACTCCAGTTTAAGAGCTATGCTGGAAACA	Gene-specific forward primer
C9E_zGfap_3	ATTTAGGTGACACTATAGGTGCCGAATCTCTTTCGGTGTTTAAGAGCTATGCTGGAAACA	Gene-specific forward primer
C9 constant	AAAAGCACCGACTCGGTGCCACTTTTTCAAGTTGATAACGGACTAGCCTTATTTTAACTTGCTATTTC TAGCTCTAAAAC	Anneal/fill-in reverse primer
C9E constant	AAAAAAAGCACCGACTCGGTGCCACTTTTTCAAGTTGATAACGGACTAGCCTTATTTAAACTTGCTAT GCTGTTTCCAGCATAGCTCTTAAAC	Anneal/fill-in reverse primer

Biology Open • Supplementary information

Table S3. PCR and cloning primers used in this study.

Primer name	Sequence (5' to 3')	Note
tyr_newF2	TCTCACTCTCCTCGACTCTTC	1st PCR for detecting attP KI at the *tyr_1* site
tyr_R2	GTAGTTTCCGGCGCACTGGCAG	
tyr-attp_F1	TGAGAGAACTCAAAGGTTACC	2nd PCR for detecting attP KI at the *tyr_1* site
tyr_R8	CCTCGACCTGACTGGACGCC	
tyr_newF2	TCTCACTCTCCTCGACTCTTC	PCR for next-generation sequencing to detect attP KI at the *tyr_1site*
tyr_R8	CCTCGACCTGACTGGACGCC	
tyr2_F1	GTCAGGTCGAGGGTTCTGTC	1st PCR for detecting attP KI at the *tyr_2* site
tyr2_R1	CGGTAGTGGACAGCTGGAAT	
tyr-attp_R1	CCAACTGGGGTAACCTTTGA	2nd PCR for detecting attP KI at the *tyr_2* site
tyr2_R2	AAGAAGCCGAACTTGCATTC	
tyr2_F1	GTCAGGTCGAGGGTTCTGTC	PCR for next-generation sequencing to detect attP KI at the *tyr_2* site
tyr2_R2	AAGAAGCCGAACTTGCATTC	
zKcnh6a-M13f	TGTAAAACGACGGCCAGTCTGTTTCCTCCACAATGCGC	1st PCR for detecting attP KI at the *kcnh6a* site
zKcnh6a-r	CTAGCCTACAGCATTTCTGAG	
zKcnh6a-2f	GCCGTCCTTGAACGAGACTG	2nd PCR for detecting attP KI at the *kcnh6a* site
zKcnh6a-2r	ATATAAACTGCCTTTTAACCGAAAT	
zGFAP-M13f	TGTAAAACGACGGCCAGTCAATTGCGGGCTCACAAACAC	1st PCR for detecting attP KI at the *gfap* site
zGFAP-r	GCTGCTGTGGAGGGACAGGC	
zGFAP-2f	CCCCCTCGGCCTTTGCCCTC	2nd PCR for detecting attP KI at the *gfap* site
zGFAP-2r	TGCTGTGACGGCTGGTCACC	
tyr_newF2	TCTCACTCTCCTCGACTCTTC	PCR for decting attL at the *tyr_1* site
attL1_RS	GAAATTTGTGATGCTATTGC	
attR1_FS	CAGGAAACAGCTATGACCATG	PCR for decting attR at the *tyr_1* site
tyr_R2	GTAGTTTCCGGCGCACTGGCAG	
zGFAP-2f	CCCCCTCGGCCTTTGCCCTC	PCR for decting attL at the *gfap* site
attL-r	AGATGAACTTCAGGGTCAGCTTGC	
M13F	GTAAAACGACGGCCAGTG	PCR for decting attR at the *gfap* site
zGFAP-r	GCTGCTGTGGAGGGACAGGC	
mCherry_F	ATAAATTCATGATGGTGAGCAAGGGCGAG	For amplifying the mcherry sequence
mCherry_R	TGAATGCAATTGTTGTTGTTAACTTG	
EcoRI-T7	AATTTAATACGACTCACTATAGGA	For introducing T7 promoter sequence
T7-EcoRI	AATTTCCTATAGTGAGTCGTATTA	
attB-P2A-EGFP-F	GATGGGTGAGGTGGAGTACGCGCCCGGGGAGCCCAAGGGCACGCCC TGGCACCCGCACCGCGGCTTCGAGgtgctacaaacttctctctgct caagcaggctggagatgtggaagaaaaccctgggcccGTGAGCAAG GGCGAGGAGCTG	1st PCR for amplifing EGFP coding sequence
pA-r	AACTTGTTTATTGCAGCTTATAATG	
attB-P2A-EGFP-F1	GATGGGTGAGGTGGAGTACGCGCCCGGGGAGCCCAAGGGCACGCC	2nd PCR for amplify the attB-P2A-EGFP sequence
pA-r	AACTTGTTTATTGCAGCTTATAATG	
6FAM-M13F	6FAM-TGTAAAACGACGGCCAGT	For PCR-FFL analysis

Number	PCR primer pair	Note
1	mCherry_F and mCherry_R	For plasmid pDestattB_ubi:mCherry construction
2	attB-P2A-EGFP-F,attB-P2A-EGFP-1 and pA-r	For plasmid pGMT-attB-P2A-EGFP construction
3	EcoRI_T7 and T7_EcoRI	For plasmid pT7_pPhC31o construction
4	tyr_newF2 and tyr_R8	For next-generation sequencing to detect attP KI at the tyr_1 site
5	tyr_newF2 and tyr_R2	For Sanger sequencing to detect attP KI at the tyr_1 site
6	tyr2_F1 and tyr2_R2	For next-generation sequencing to detect attP KI at the tyr_2 site
7	tyr2_F1 and tyr2_R1	For Sanger sequencing to detect attP KI at the tyr_2 site
8	zGFAP-2f and zGFAP-2r	For next-generation sequencing to detect attP KI at the gfap site
9	zGFAP-M13f and zGFAP-2r	For Sanger sequencing to detect attP KI at the gfap site
10	zKcnh6a-2f and zKcnh6a-2r	For next-generation sequencing to detect attP KI at the Kcnh6a site
11	zKcnh6a-M13f and zKcnh6a-2r	For Sanger sequencing to detect attP KI at the Kcnh6a site
12	tyr_newF2 and attL1_RS	PCR for decting attL at the tyr_1 site
13	attR1_FS and tyr_R2	PCR for decting attR at the tyr_1 site
13	zGFAP-2f and attL-r	PCR for decting attL at the gfap site
15	M13F and zGFAP-r	PCR for decting attR at the gfap site

Table S4. Sequences of the single-stranded oligonucleotides (ssODNs) used for attP knock-in experiments. The ssODNs were chemically synthesized and two phosphorothioate linkages (denoted by asterisks) were added to both termini to enhance stability. The knock-in sequences are shown in bold. The attP sequences are shown in bold uppercase letters. The bold lowercase letters were added to avoid stop codons in the reading frames.

Name	Sequence (5' to 3')
tyr1-attP-KI	T*T*CATCATCATGTCTCTCCATCTCCTCCTCTTCTTCTTCCTCCAGCTCTTCAGCTCGTCTCTCCAGCAGTTCCCCC GAGTCTGCACCT**CCCCCAACTGAGAGAACTCAAAGGTTACCCCAGTTGGGG**CAGAAGTCCTCCAGTCCAAACGCTGCT GTCCAGT*C*T
tyr2-attP-KI	G*C*CGCAATCAAACCCCATGTAGTTTCCGGCGCACTGGCAGGTTTGGTTGTAAAACACTAAAGGCCATCGCTCTCGA TCGTCCACTCCTGAG**CCCCCAACTGAGAGAACTCAAAGGTTACCCCAGTTGGGG**TGAGGATACTGCGGCCCGTTGGGA AGGTCGGACA*C*C
gfap-attP-KI	A*T*CAGACCCCCCCTCTCTCTTTCACCCCCCACATCAACTCTATAAAAACCCAGAGTTCACCCGCACTCGATCTCAT TCTCCTCCACCATGG**agCCCCCAACTGAGAGAACTCAAAGGTTACCCCAGTTGGGGg**AGTCCCAGCGTTCCTTCTCAT CCTACCGAAAGAG*A*T
kcnh6a-attP-KI	a*g*acatgaaaaagttggtaaactcacTTTGCCCGTCGAATTTCCTAATGATGGTGTCCAGATAGGTGTTCTGGAGA GCGACATGTCCGCG**tctCCCCAACTGGGGTAACCTTTGAGTTCTCTCAGTTGGGGGa**CGCACGGGCATTTTTCACGCG TGGACAGATTCAA*G*A

Table S5. Fluorescent PCR fragment length analysis for gRNA efficiency. For each gRNA, two pools of embryos (10 embryos per pool) were analyzed. Samples K5, K6, G7, and G8 are uninjected control embryos. PCR products were run on ABI 3730xl DNA Analyzer at MGH DNA core. Fragment sizes, peak heights, and peak areas were determined using GeneMapper v4.0. The fragments correspond to the wild-type alleles are highlighted in yellow. gRNA efficiencies were determined by comparing the abundance of the wild-type alleles over the sum of all peaks in the same samples based on peak intensity. For attP knock-in, kcnh6a_2 and gfap_1 gRNAs were used.

Sample Name	gRNA	Size 1	Size 2	Size 3	Size 4	Size 5	Size 6	Size 7	Size 8	Size 9	Height 1	Height 2	Height 3	Height 4	Height 5	Height 6	Height 7	Height 8	Height 9	Peak Area 1	Peak Area 2	Peak Area 3	Peak Area 4	Peak Area 5	Peak Area 6	Peak Area 7	Peak Area 8	Peak Area 9
K1	kcnh6a_1	286.59									9269									105184								
K2	kcnh6a_1	286.59									10777									120346								
K3	kcnh6a_2	280.17	282.13	286.68							1242	862	4446							12559	8309	47710						
K4	kcnh6a_2	280.22	282.12	286.65							2021	1456	3728							20934	13569	39176						
K5	-	286.71									9192									100197								
K6	-	286.69									10088									111595								
G1	gfap_1	246.18	254.89	264.28							127	302	1152							1437	2886	11533						
G2	gfap_1	243.03	255.05	257.96	260.66	264.44	280.25				80	659	186	757	323	167				700	6968	1669	7948	2903	1798			
G3	gfap_2	235.1	238.64	250.11	252.89	254.82	257.6	262.3	265.07	272.9	62	74	240	305	256	115	582	91	68	1010	592	2493	3288	2405	995	6105	951	1021
G4	gfap_2	231.08	242.96	250	254.67	261.2	264.95	269.75	278.18		212	646	178	981	591	308	221	362		2207	6682	1775	10592	6482	3157	2158	4126	
G5	gfap_3	263.99									4402									47518								
G6	gfap_3	264									4310									46587								
G7	-	264.08									5391									61207								
G8	-	264.02									5805									66142								
